# Stabilized perovskite ink for scalable coating enables high-efficiency perovskite modules

**DOI:** 10.1126/sciadv.aec0915

**Published:** 2026-01-02

**Authors:** Yangyang Liu, Junke Wang, Tianxiao Liu, Lingyuan Wang, Yuhan Zhou, Yaoyao Zhang, Yunjie Dou, Xiaoyu Shi, He Yan, Akash Dasgupta, Henry J. Snaith, Shangshang Chen

**Affiliations:** ^1^ State Key Laboratory of Coordination Chemistry, MOE Key Laboratory of High-Performance Polymer Materials & Technology, School of Chemistry and Chemical Engineering, Nanjing University, Nanjing 210023, China.; ^2^ Clarendon Laboratory, Department of Physics, University of Oxford, Parks Road, Oxford OX1 3PU, UK.; ^3^ Department of Chemistry, Hong Kong University of Science and Technology, Kowloon 999077, Hong Kong.

## Abstract

Perovskite inks play critical roles in determining film quality and device performance, and ink stability is desired to ensure high device reproducibility. Here, we reveal the instability issue of current cesium-formamidinium lead triiodide (Cs*_x_*FA_1−*x*_PbI_3_) inks whose aggregation and precipitation tendencies are induced by excessively strong solvent-lead-halide coordination. By modulating coordination strength between precursor salts and solvents, we identify solvent coordination-dispersion equilibrium as the governing factor for ink stability and develop a stable ink that exhibits a remarkable increase in the shelf life. It effectively tunes ink drying and film crystallization, resulting in blade-coated perovskite films with excellent uniformity and low defect density. This enhancement led to increased aperture efficiency of ambient-fabricated p-i-n perovskite modules to 23.5%. The resultant devices also exhibit high durability, and 99% of the initial PCE was retained after 1700 hours of maximum power point tracking following the ISOS-L-2 standard protocol.

## Teaser

The critical effects of solvent coordination on the stability of perovskite inks.

## INTRODUCTION

Perovskite solar cells (PSCs) have emerged as a promising technology for next-generation photovoltaics due to their exceptional power conversion efficiencies (PCEs) and predicted low manufacturing costs (*1*, *2*). The solution-processable nature of perovskites enables large-scale production of solar panels, making them a compelling alternative to traditional silicon-based cells (*3*–*6*). As anticipated, the composition of the perovskite inks (or precursor solutions), a critical component in the solution fabrication process, substantially influences the final performance of solar devices (*7*).

The precursor solution consists of a mixture of organic and inorganic salts dissolved in a suitable solvent. Upon film casting and solvent evaporation, the components form the perovskite crystal structure, responsible for efficient light absorption and conversion (*5*, *8*). The choice of A-site cations [methylammonium (MA), formamidinium (FA), or cesium (Cs)], inorganic halide anions (iodide or bromide), their stoichiometric ratio, and the solvent all play critical roles in determining the perovskite film morphology, crystallinity, and defect density (*9*–*13*). Since the thermal instability of MA cations has been reported multiple times (*14*), the Cs*_x_*FA_1−*x*_PbI_3_ composition has gained popularity in spin-coated cells due to its thermal stability and narrow bandgap for sufficient light harvesting (*15*, *16*).

When transitioning such a promising composition to scalable production via blade or slot-die coatings, the solvent system used to dissolve the precursor salts is essential. It not only affects the solution’s viscosity, drying rate, and the formation of perovskite films but also affects the stability and shelf life of the precursor solution (*5*, *7*). Recently, one of the prevalent Cs*_x_*FA_1−*x*_PbI_3_ precursor solutions for large-area coating is dissolving the corresponding salts in a 2-methoxyethanol (2-ME)/dimethyl sulfoxide (DMSO) mixture (*17*, *18*). DMSO coordinates with Pb cations, slowing the crystallization of the perovskite film, and was able to realize a certified module PCE of 21.8% assisted with interface passivation (*19*). Although this precursor solution has resulted in multiple reports of high-efficiency perovskite modules, its shelf life has not been studied and whether it is suitable for real-world module manufacturing remains an open question. The shelf life of perovskite inks is crucial for the long-term performance and reliability of perovskite devices, and stable inks ensure consistent film formation during the scalable deposition process, leading to devices with uniform morphology and reduced variability (*20*).

In this work, we reveal the instability issue of previous perovskite inks including 2-ME/DMSO due to strong coordination-induced aggregation and precipitation. This greatly limits its shelf life to less than 15 min, making it challenging for real-world scalable fabrication. The instability and aggregation also hinder reproducibility and can be expected to clog tubes in a continuous coating line. To address these issues, we modulate the coordination strength and solvent solvability and identify solvent coordination-dispersion equilibrium as the governing factor for ink stability. Based on our quantitative analysis, we developed a stable perovskite precursor solution using cesium iodide (CsI), formamidinium iodide (FAI), and PbI_2_, dissolved in *N*,*N*-dimethylformamide (DMF)/*N*-methyl-2-pyrrolidone (NMP). This ink exhibits a shelf life of up to over 10,000 min in air, which is also much longer than a similar solvent system previously used with MAI and PbI_2_ (*21*). This improvement is attributed to the moderate coordination strength between NMP and the precursor salts, and reduced aggregation and precipitation compared to the conventional 2-ME/DMSO case. Furthermore, the DMF/NMP (5.2:1, v:v) precursor solution effectively tunes ink drying and film crystallization (*21*), resulting in blade-coated perovskite films with excellent uniformity and low defect density fabricated in ambient conditions. This enhancement led to increased aperture PCEs of blade-coated p-i-n perovskite mini-modules to 23.5% (independently certified as 22.84%). The resultant encapsulated cells also exhibit exceptional operational stability tested following the ISOS-L-2 standard protocol at 65°C in air, and 99% of the initial PCE was retained after 1700 hours of continuous light soaking.

## RESULTS

**Fig. 1.**
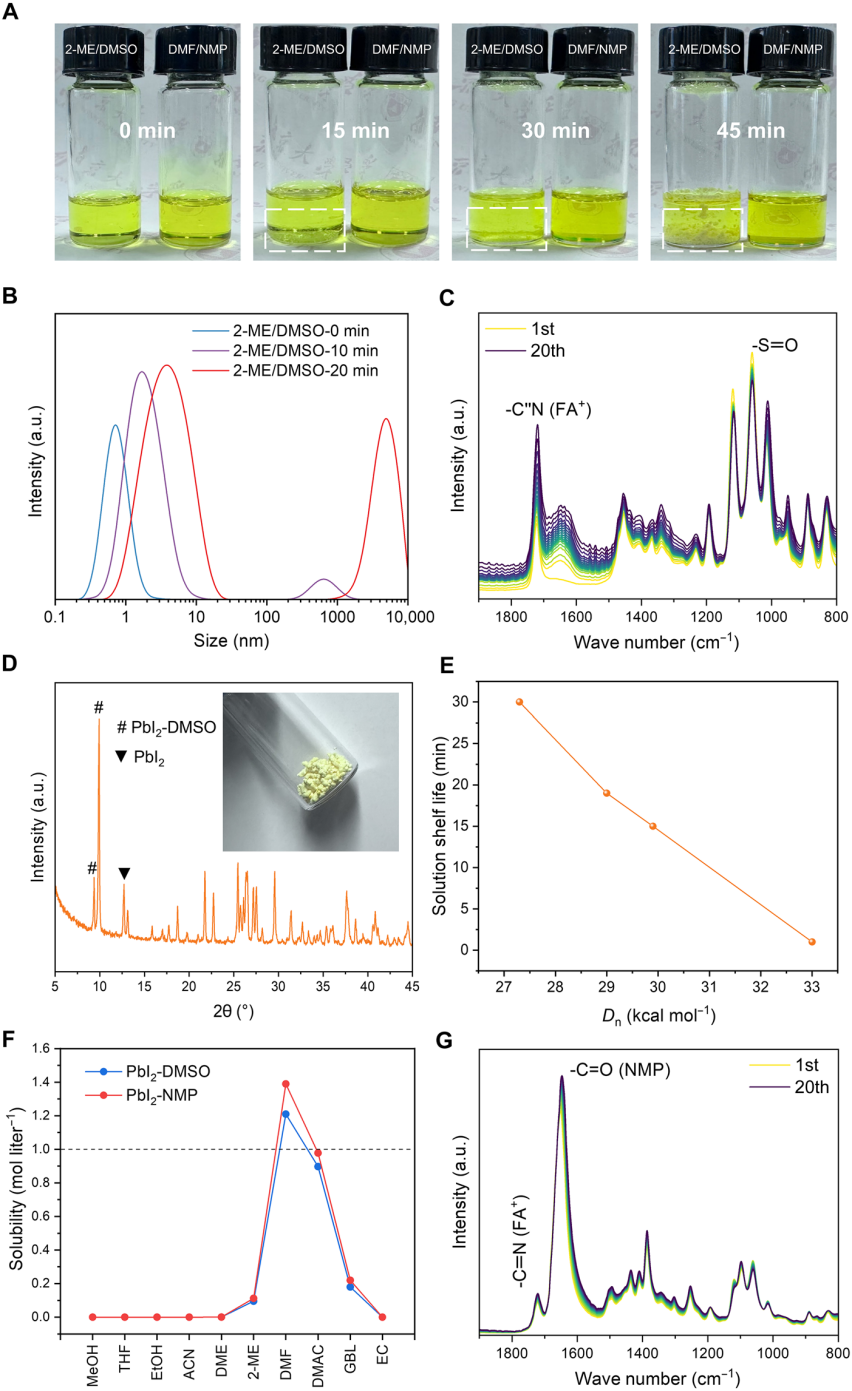
Stability of perovskite precursor solutions. (**A**) Photographs of Cs_0.1_FA_0.9_PbI_3_ in 2-ME/DMSO (left) and DMF/NMP (right) solvents stored in air (25°C, 50% RH) for 45 min. (**B**) DLS spectra of the 2-ME/DMSO ink after different storage durations. (**C**) In situ FTIR measurement of the 2-ME/DMSO ink in air. (**D**) XRD patterns of the precipitate isolated from aged Cs_0.1_FA_0.9_PbI_3_ 2-ME/DMSO ink. Inset shows the photograph of the isolated precipitate. (**E**) Ink shelf time versus *D*_n_ of coordinating solvents in the 2-ME–based Cs_0.1_FA_0.9_PbI_3_ perovskite solutions. (**F**) Solubilities of (PbI_2_-DMSO) and (PbI_2_-NMP) precipitates in a variety of noncoordinating solvents (methanol, MeOH; tetrahydrofuran, THF; ethanol, EtOH; acetonitrile, ACN; 1,2-dimethoxyethane, DME; *N*,*N*-dimethylacetamide, DMAC; γ-butyrolactone, GBL; ethylene carbonate, EC). (**G**) In situ FTIR measurement of the DMF/NMP ink in air. a.u., arbitrary unit.

The shelf life of perovskite precursor solutions is a critical factor for ensuring high device reproducibility and fabrication efficiency (*6*, *22*). We initially examined the intrinsic stability of a widely used Cs_0.1_FA_0.9_PbI_3_ 2-ME/DMSO solution in ambient [25°C and 50% relative humidity (RH)]. In this solution, the nonvolatile DMSO solvent with a donor number (*D*_n_) of 29.9 kcal mol^−1^ coordinates with Pb, slowing down perovskite crystallization, especially for FA-dominant compositions whose crystallization is even faster and harder to control. As shown in Fig. 1A, the 2-ME/DMSO precursor solution (left) initially appeared clear but formed small white precipitates after 15 min. Within 45 min, large aggregates accumulated at the bottom of the vial, indicating notable phase separation.

We further used dynamic light scattering (DLS) measurement to investigate the colloid size and microcrystal formation in the 2-ME/DMSO perovskite precursor solution (Fig. 1B). The DLS curve of the pristine Cs_0.1_FA_0.9_PbI_3_ solution in 2-ME/DMSO showed a single peak at 0.7 nm, indicating minimal nucleation or growth of microcrystals. After storage in air for 10 min, two distinct peaks emerged at 2 and 600 nm, suggesting the formation of microcrystals with uncontrolled dimensions (*23*). After 20 min, the DLS curve revealed a remarkable shift in the peaks, indicating the gradual growth and size increase of the microcrystals, consistent with our experimental observations (Fig. 1A). Meanwhile, the in situ Fourier transform infrared (FTIR) spectroscopy spectrum revealed a notable change in the ─S═O peak in the 2-ME/DMSO solution during the storage in air (Fig. 1C and fig. S1), indicating its strong coordination tendency to Pb, resulting in the large aggregate and precipitate formed. To identify the composition of the precipitate, it was carefully isolated from the 2-ME/DMSO perovskite ink. The x-ray diffraction (XRD) analysis of the precipitate is presented in Fig. 1D. The diffraction pattern reveals prominent peaks at 9.4° and 9.9°, which are indexed to a PbI_2_-DMSO complex (*24*), and a less intense peak at 12.7°, characteristic of PbI_2_. These findings substantiate the hypothesis that the coordination of DMSO to Pb^2+^ is the primary cause of precipitation, thereby diminishing the long-term stability of the perovskite ink. In addition to tuning the perovskite crystallization dynamics, we unveil that the PbI_2_-DMSO complex also has a critical effect on the shelf life of perovskite inks.

To further investigate the impact of solvent coordination strength on perovskite ink stability, we systematically studied inks prepared with 2-ME and various coordinating solvents: 1,3-dimethyl-3,4,5,6-tetrahydro-2(1*H*)-pyrimidinone (DMPU; *D*_n_: 33.0 kcal mol^−1^), 1,3-dimethyl-2-imidazolidinone (DMI; *D*_n_: 29.0 kcal mol^−1^), and *N*-methyl-2-pyrrolidone (NMP; *D*_n_: 27.3 kcal mol^−1^) (*25*, *26*). A higher *D*_n_ indicates stronger coordination between the solvent and Pb cations. As shown in fig. S2, the storage stability of the perovskite inks is directly correlated with the *D*_n_ of the coordination solvent. The perovskite ink based on DMPU, with the highest *D*_n_, exhibited rapid precipitation within 1 min. In contrast, NMP-based ink showed precipitation after 30 min. We plot the shelf life of the ink versus the *D*_n_ of the coordination solvents in Fig. 1E, and it exhibits a clear linear relationship with the *D*_n_ of the coordinating solvents. To extend the ink shelf life, it is crucial to balance the coordination strength of the coordinating solvents, slowing down perovskite crystallization without inducing excessive aggregation.

Based on these observations, we identify solvent coordination-dispersion equilibrium as the governing factor for ink stability and propose a critical criterion for stabilizing perovskite inks: The coordinate compound formed between the coordinating solvent and Pb cations must be effectively dispersed by the noncoordinating solvent at thermodynamic equilibrium. To determine the solubilities of the PbI_2_-DMSO precipitate at thermodynamic equilibrium, we further dissolved it in commonly used noncoordinating solvents and measured their maximum solubilities (calculated based on PbI_2_-DMSO adduct) at room temperature. Given that perovskite inks typically require ABX_3_ concentrations exceeding 1 mol liter^−1^, our investigation (as shown in Fig. 1F) revealed that only DMF achieved a solubility greater than 1 mol liter^−1^ in a thermally stable state. Conversely, while other solvents initially dissolved moderate amounts of perovskite precursor salts, they demonstrated limited capacity for dissolving the Pb-coordinating PbI_2_-DMSO compound under thermodynamic equilibrium conditions. We also quantified the solubilities of the PbI_2_-NMP compound in these solvents, and a similar trend was observed. Crucially, the weaker coordination strength of NMP resulted in higher solubilities for the PbI_2_-NMP intermediate compound compared to PbI_2_-DMSO complexes. This is exemplified by its maximum solubility in DMF, which approached 1.4 mol liter^−1^, thereby offering a broader window for concentration adjustment.

In this case, we propose using the DMF/NMP mixture as the solvent. NMP can coordinate with Pb without forming excessive aggregation due to its smaller *D*_n_ than DMSO. In addition, DMF has a higher solvability for the intermediate compound and also a lower boiling point, making it easier to evaporate during scalable coating. As shown in Fig. 1A, the DMF/NMP precursor solution (right) exhibited much improved stability with no notable precipitation after 45 min (Fig. 1A). The DLS curve for the DMF/NMP precursor solution revealed a single peak (fig. S3), remaining relatively unchanged over 20 min. This result indicates a well-controlled colloidal system with a relatively narrow particle size distribution, promoting uniform nucleation and growth of perovskite microcrystals. In situ FTIR measurements showed almost no changes in the ─C═O peak (from the NMP molecule) at 1648 cm^−1^ in the DMF/NMP solution during 20 scans at the same time intervals (Fig. 1G) (*13*, *27*).

We further study the effects of solvent ratios on the stability of perovskite precursor solutions. A series of Cs_0.1_FA_0.9_PbI_3_ precursor solutions were prepared by varying the volume ratio from 9:1 to 0:10, and their storage stability was monitored for 48 hours under ambient conditions (25°C, 50% RH). We observed pronounced differences between the two types of inks. The corresponding images (figs. S4 and S5) demonstrate that in the 2-ME/DMSO system, increasing the DMSO content greatly accelerates precipitation and aggregation. For instance, the ink containing 60% DMSO exhibited noticeable turbidity and sedimentation much earlier than that with only 10% DMSO. Even at DMSO contents as high as 80 to 90%, some degree of precipitation persisted. In contrast, all DMF/NMP solutions exhibited markedly enhanced stability. Even solutions with high NMP content remained optically clear and well-dispersed for extended durations.

This contrast between the two systems provides compelling evidence for the wide tunability in the DMF/NMP solvent system. The moderate coordination strength of NMP (relative to DMSO), combined with the high solvability of DMF, facilitates effective dispersion of the PbI_2_-solvent complexes, thereby preventing excessive aggregation. These results further support our proposed mechanistic model: Achieving an optimal balance between solvent coordination (to slow down perovskite crystallization) and dispersion (to prevent aggregation) is essential for maintaining long-term precursor solution stability.

Perovskite films were fabricated using the blade-coating method in ambient conditions (25°C and 30% RH) and then thermally annealed at 150°C (5 min) and 120°C (30 min) for the perovskite films processed from 2-ME/DMSO and DMF/NMP inks, respectively. The drying of the perovskite precursor solutions during blade-coating was first recorded with a metallographic microscope as a function of drying time (fig. S6). It is observed that several crystal formations occurred immediately in the pristine wet film. Notably, the crystal formation in the wet film derived from the DMF/NMP precursor solution was found to be more uniform than that observed in the 2-ME/DMSO sample. This indicates that the DMF/NMP precursor solution effectively suppresses rapid natural supersaturation and uncontrolled nucleation and grain growth processes, ultimately resulting in a condensed perovskite film with a larger grain size (figs. S7 to S10) (*28*–*30*). This phenomenon can be attributed to the moderate interactions between the DMF/NMP solvent system and the perovskite precursor salts.

**Fig. 2.**
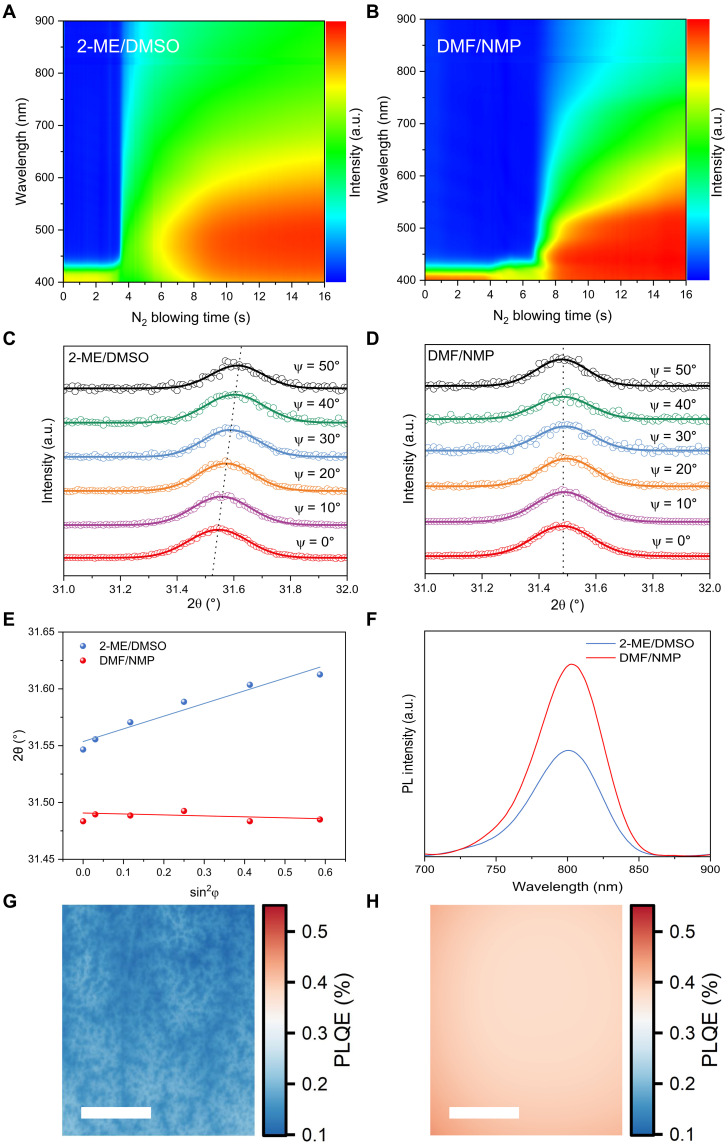
Film characterization. In situ UV-vis film absorption spectra over time for (**A**) 2-ME/DMSO and (**B**) DMF/NMP samples during blade coating. Grazing incidence XRD (GIXRD) patterns at different tilt angles for (**C**) 2-ME/DMSO and (**D**) DMF/NMP perovskite films. (**E**) Linear fit of 2θ-sin^2^φ in the GIXRD patterns. (**F**) PL spectra of the 2-ME/DMSO and DMF/NMP perovskite films deposited on glass substrates (excited from the top surface of perovskite films at a wavelength of 485 nm, ~60 mW cm^−2^). Luminescence imaging under approximately one sun photoexcitation with a blue (465 nm) light-emitting diode of (**G**) 2-ME/DMSO and (**H**) DMF/NMP perovskite films. Scale bars, 1 mm. PLQE, photoluminescence quantum efficiency.

We further investigated the nucleation and crystallization of perovskite during the blade-coating process using in situ ultraviolet-visible (UV-vis) absorption measurements. A schematic of the in situ optical measurement system is presented in fig. S11. Figure 2 (A and B) displays the two-dimensional contour plots of the UV-vis spectra for the 2-ME/DMSO and DMF/NMP precursor solutions during the blade-coating process. In situ UV-vis absorption spectra (where 0 s on the time axis corresponds to the onset of air knife blowing) show that the fabricated perovskite wet films exhibit characteristic absorption signals of precursor ions and colloidal complexes in the wavelength range below 450 nm (*3*, *31*–*33*). Following air blowing, the solvent evaporation led to an increase in solute concentration and enhanced light absorption. The abrupt rise in absorption toward longer wavelength indicates the formation of a majority of intermediate phases. By monitoring the optical absorbance at 450 nm (fig. S12A), it was observed that the intermediate phase formed markedly faster in the 2-ME/DMSO ink than in the DMF/NMP system. This suggests that intermediate phase formation is thermodynamically more favorable in the 2-ME/DMSO case.

In the 2-ME/DMSO solvent system, the saturated vapor pressure of 2-ME is 1.3 kPa at 20°C, which rapidly escapes within 3.5 s after air blowing, while the saturated vapor pressure of DMSO is 0.049 kPa at 20°C and remains within the wet film during the blowing process. In addition, DMSO exhibits a strong binding affinity with PbI_2_, resulting in a notable absorption peak appearing only 3 s after the rapid escape of 2-ME. This delay in the evaporation of DMSO solvent molecules from the wet film may lead to an uneven nucleation rate in both bulk and surface, ultimately resulting in poorer crystallization quality. In the DMF (0.36 kPa)/NMP (0.038 kPa) solvent system, the primary solvent DMF has a slower evaporation rate, resulting in a longer solvent extraction window. After 6.8 s of blowing, the absorption begins to shift toward the longer wavelength. Meanwhile, the moderate binding energy of NMP with PbI_2_ leads to a rapid appearance of a strong absorption peak (near 450 nm) in just 1 s after solvent escaping, indicating a more uniform nucleation rate of PbI_2_-NMP intermediate phase in both the bulk and surface, which facilitates the acquisition of high-quality perovskite films.

We further used the Avrami model to analyze the crystallization rate and dimensionality of the blade-coated perovskite (*34*, *35*). The detailed model can be described as follows

$$f\left(t\right)=1-e^{\left[-K{\left(t-t_{0}\right)}^{n}\right]}$$

Here, *f*(*t*) represents the crystallized volume fraction, *K* is the crystallization rate constant, and *n* is the Avrami index (*35*, *36*). To eliminate the effects of intensity variations caused by air scattering, *f*(*t*) is defined as the difference between the absorption shoulder intensity at 720 nm and the cutoff absorption intensity at 760 nm of the precursor film at the end of the blade coating (fig. S12B). As shown in fig. S12 (C and D), the crystallization rate constant *K* decreased from 0.101 to 0.0045 s^−1^ for the 2-ME/DMSO and DMF/NMP systems respectively, which can be attributed to the optimization of the intermediate phase facilitated by precursor solution modulation. Notably, this optimization also resulted in a change in the crystallization mechanism of the perovskite. The Avrami index *n* reflects the nucleation mode and the dimensionality during perovskite crystallization. The 2-ME/DMSO film exhibited an *n* value of 1.63, while the DMF/NMP film displayed two-dimensional growth with a larger *n* value of 2.21. This indicates that nucleation in the 2-ME/DMSO film primarily occurs in one direction, leading to rapid extension of the crystals along a specific direction. In contrast, nucleation in the DMF/NMP film occurred simultaneously in multiple directions, facilitating the formation of a more uniform layered structure. This phenomenon is primarily attributed to the relatively lower binding energy between NMP and PbI_2_ octahedra in comparison to DMSO. The weaker coordination interaction of NMP with PbI_2_ enables more controllable nucleation and subsequent crystallization processes in the DMF/NMP-based precursor film following the application of air knife blowing. Under identical blowing conditions (0.3 MPa), the nucleation barrier of the PbI_2_-DMSO intermediate compound in the 2-ME/DMSO solvent system is considerably higher, primarily due to the stronger binding affinity of DMSO with PbI_2_. In contrast, the moderate binding energy between NMP and PbI_2_ in the PbI_2_-NMP intermediate compound results in a reduced nucleation barrier. This facilitates regulated nucleation events upon blowing, promoting the controlled nucleation and crystallization with enhanced crystallinity and improved film morphology. The above interpretations are consistent with and supported by the optical microscopy observations presented in fig. S6, which visually corroborate the differences in nucleation behavior and film uniformity between the two solvent systems.

We further quantitatively assessed the longitudinal uniformity and lattice mismatch of perovskite films prepared from two different precursor solutions using grazing incidence XRD residual strain analysis. Given that the (012) crystal plane exhibits a high diffraction angle and a multiplicative factor that provides more reliable structural symmetry information, we selected this plane for further analysis (Fig. 2, C and D). At a fixed depth, the diffraction data were fitted using a Gaussian distribution function, revealing a shift of the diffraction peak position toward lower 2θ values with increasing penetration depth. Typically, the relationship between sin^2^φ and 2θ is linear, with the slope of the fitted line representing the magnitude of the residual strain. As shown in Fig. 2E, the fitted slope for the films prepared from the 2-ME/DMSO precursor solution is positive, indicating that the (012) crystal plane exhibits compressive strain when oriented out of the plane at the same detection depth (*37*). This observation is consistent with the presence of pinholes noted in the resulting 2-ME/DMSO films. In contrast, the fitted line for the perovskite films derived from the DMF/NMP precursor solution demonstrates a smaller absolute slope, suggesting a minimal residual compressive strain. We also investigated the compositional inhomogeneity via time-of-flight–secondary ion mass spectrometry (TOF-SIMS). As shown in fig. S13, the 2-ME/DMSO film exhibits a gradual increase in Cs^+^ signal intensity with sputtering time, indicating a top-to-bottom gradient of Cs^+^ enrichment. In contrast, the DMF/NMP film demonstrates a more uniform Cs^+^ distribution profile. Such a difference is probably attributed to the distinct nucleation orientation shown in fig. S12. The nucleation in the 2-ME/DMSO system proceeds predominantly in a single direction, promoting rapid crystal growth along a specific axis. In contrast, the nucleation in the DMF/NMP system occurs simultaneously along multiple directions, thereby facilitating the development of a more uniform perovskite structure with improved homogeneity in Cs^+^ distribution. These results support the vertical Cs^+^ inhomogeneity also contributed to the observed 2θ shifts in Fig. 2, C and E.

The photoluminescence (PL) properties of the perovskite films were then investigated. Compared to the film prepared from the 2-ME/DMSO ink, the perovskite film prepared with the DMF/NMP precursor solution exhibited an 81% increase in PL intensity (Fig. 2F), confirming the suppressed nonradiative recombination in the perovskite films fabricated from the DMF/NMP precursor solution. Meanwhile, we used luminescence imaging to investigate macroscopic inhomogeneities within the perovskite films (*38*). As shown in Fig. 2 (G and H), the perovskite film fabricated using the DMF/NMP ink displayed superior uniformity with much enhanced PL intensity, while the 2-ME/DMSO film exhibited poor uniformity and distinct island-like dark regions.

We also studied the impact of ink aging on the crystallinity and morphology of blade-coated perovskite films. XRD patterns provide direct evidence of the chemical and structural evolution in films derived from aged 2-ME/DMSO ink (fig. S14). A distinct PbI_2_ peak emerges at 2θ ≈ 12.5° after 45 min of aging, indicating substantial chemical degradation of the perovskite precursors. This prominent PbI_2_ signal originates from the thermally stable PbI_2_-DMSO intermediate complex, which releases DMSO upon thermal annealing without converting to the perovskite phase. These results demonstrate that ink aging not only modifies aggregation states but also induces fundamental chemical changes that directly affect the final film composition. In contrast, films processed from DMF/NMP ink showed no notable shifts in peak positions or variations in diffraction intensity. Scanning electron microscopy (SEM) characterization further revealed the evolution of surface morphology in the perovskite films (fig. S15). For the 2-ME/DMSO system, prolonged ink aging led to the gradual development of pinholes and alterations in grain surface morphology. In addition, needle-like PbI_2_ crystals emerged on the film surface, consistent with the chemical degradation indicated by XRD. Conversely, the DMF/NMP-based films exhibited no much morphological changes or pinhole formation, even after extended aging.

To evaluate the photovoltaic performance of PSCs fabricated using two distinct solvent systems, we constructed devices with a p-i-n architecture of glass/indium-doped tin oxide (ITO)/poly[bis(4-phenyl)(2,4,6-trimethylphenyl)amine (PTAA)/Cs_0.1_FA_0.9_PbI_3_/C_60_/bathocuproine (BCP)/copper (Cu). Both PTAA hole transporter and perovskite films were prepared by the blade coating method under ambient conditions. Several reported additives have also been included in the perovskite inks, and we have provided a table summarizing their effects on film properties (table S1). For instance, l-α-phosphatidylcholine (LP) has been studied to facilitate the ink spreading and tune fluid dynamics during blade-coating (*39*), and 4-fluoro-phenyethylammonium iodide (p-F-PEAI) exhibits effective passivation to perovskite interfacial defects (*40*). It should be noted that these additives have negligible influence on the ink degradation observed in Fig. 1A. Even no additives, 2-ME/DMSO ink still showed similar precipitate and degraded after 15 min (fig. S16).

**Fig. 3.**
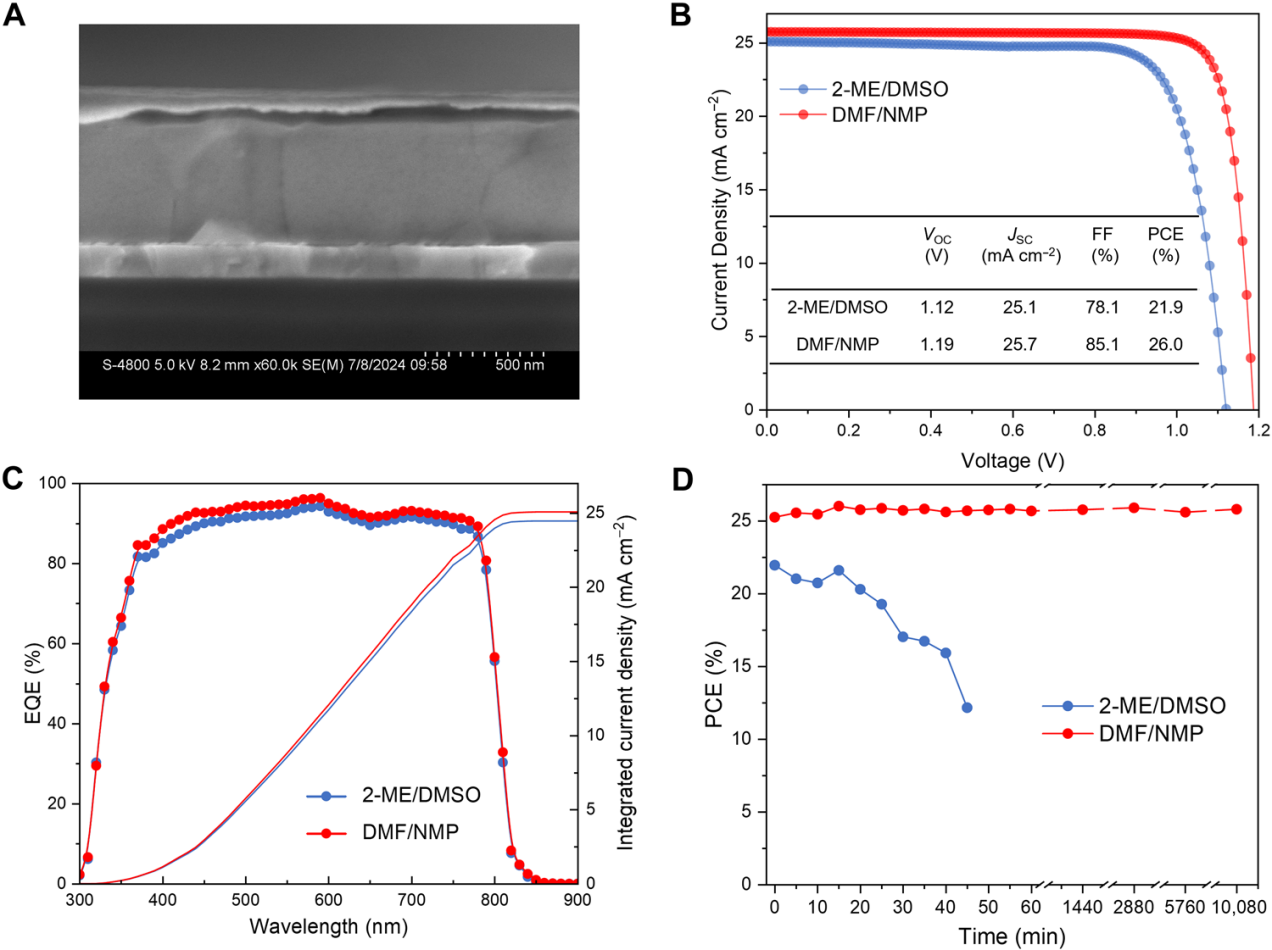
Device characterization. (**A**) Cross-sectional SEM image of the DMF/NMP PSC. (**B**) *J*-*V* characteristic curves (reverse sweeping) of champion 2-ME/DMSO and DMF/NMP devices. Inset shows the detailed photovoltaic parameters. (**C**) EQE curves of the 2-ME/DMSO and DMF/NMP devices. (**D**) Cell PCEs versus the aging time of the 2-ME/DMSO and DMF/NMP inks in the ambient conditions.

As illustrated in the cross-sectional SEM image (Fig. 3A), the perovskite layer prepared using the DMF/NMP precursor solution exhibited monolithic and uniform grains with reduced grain boundaries, which is likely to facilitate effective charge transport and reduce carrier recombination (*10*). The champion 2-ME/DMSO cell delivered an open-circuit voltage (*V*_OC_) of 1.12 V, a short-circuit current density (*J*_SC_) of 25.1 mA cm^−2^, a fill factor (FF) of 0.781, and thus a decent PCE of 21.9% (Fig. 3B), consistent with previous reports (*17*). In contrast, the use of the DMF/NMP solvent system greatly enhanced device PCE due to improved crystallinity, resulting in an optimal champion cell (6.84 mm^2^ aperture area) with a *V*_OC_ of 1.19 V, a *J*_SC_ of 25.7 mA cm^−2^, an impressive FF of 0.851, and thus a PCE more than 26% (Fig. 3B). Notably, this is the highest PCE reported for ambient-processed PSCs, regardless of fabrication methods or device structures (table S2), and is comparable to those fabricated in an inert atmosphere.

The champion cell based on the DMF/NMP ink was sent to an accredited solar device testing center for third-party certification. A stabilized power output was recorded for 300 s, and a stabilized efficiency of 26.05% was confirmed (fig. S17). The champion cell based on the DMF/NMP precursor solution also showed negligible hysteresis (fig. S18), and an average PCE of (25.6 ± 0.3)% was obtained (fig. S19). The external quantum efficiency (EQE) spectra show that DMF/NMP cells exhibit more efficient photon-to-current conversion efficiency in the range of 400 to 800 nm than reference cells (Fig. 3C). The voltage deficit in this case was minimized to 0.34 V calculated from the EQE spectrum onset of 810 nm (fig. S20). The improvement in device efficiency is primarily attributed to the enhancement of perovskite crystallinity, the reduction of voids at the bottom, and the optimization of the buried interface.

To investigate the impact of precursor solution aging on the cell PCEs, we prepared two groups of PSCs using 2-ME/DMSO and DMF/NMP precursor solutions aged for various durations. As illustrated in Fig. 3D, fig. S21, and table S3, the PCEs of perovskite films derived from the 2-ME/DMSO solvent system exhibited a strong dependency on the aging time, with a short shelf life of ~15 min. Beyond this time period, the efficiency of the resulting devices began to decline substantially. In contrast, devices based on the DMF/NMP solvent system showed considerably less time dependence, and after 10,080-min aging, a high PCE of 25.8% is maintained (table S4). During the submission of this work, we found that the resultant PCE can still reach 25.5% after 120-day storage in air. Compared to recent reports, our work represents one of the few investigations into the degradation mechanisms of inks suitable for scalable coating processes, ultimately achieving exceptional stability for large-area coating applications (table S5). These findings underscore the advantages of the DMF/NMP solvent system over 2-ME/DMSO in terms of storage stability, thereby facilitating its potential for industrial-scale application.

**Fig. 4.**
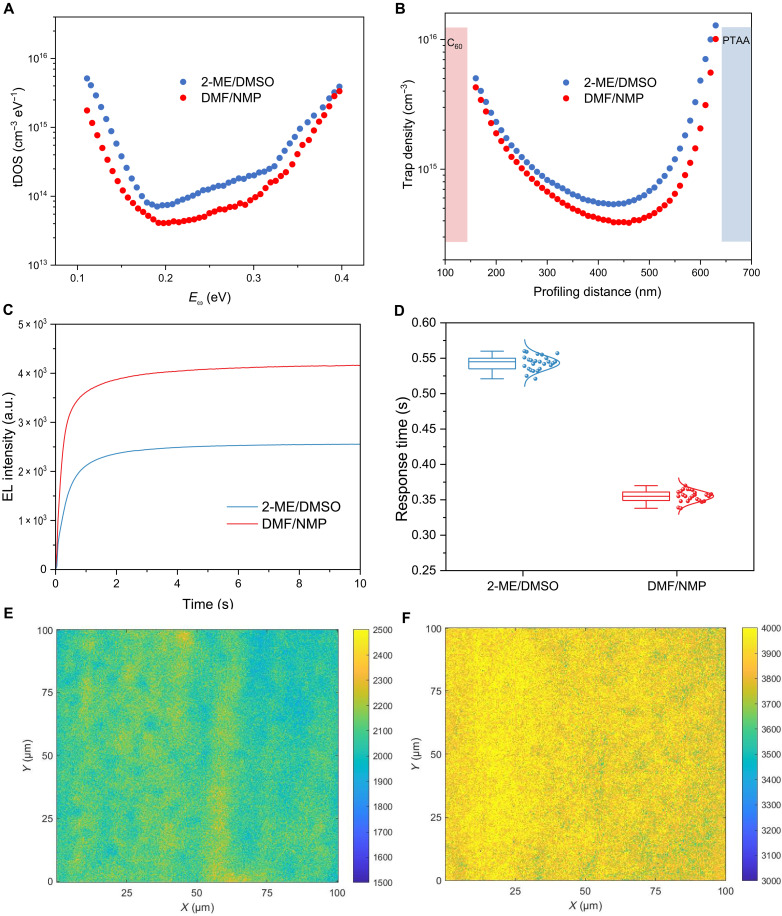
Trap and uniformity characterization. tDOS (**A**) and DLCP (**B**) spectra of 2-ME/DMSO and DMF/NMP PSCs. (**C**) EL intensity versus bias application time for 2-ME/DMSO and DMF/NMP PSCs. (**D**) Average EL response time summarized from the EL mapping results based on 25 randomly selected areas. The elements of the box plot are defined as follows: center line: median; box limits: upper and lower quartiles; whiskers: 1.5 times the interquartile range. EL intensity mapping for (**E**) 2-ME/DMSO and (**F**) DMF/NMP PSCs, respectively.

We used thermally admittance spectroscopy and drive-level capacitance profiling (DLCP) characterizations to investigate the variations in trap density within the devices. The trap density of states (tDOS) curve presented in Fig. 4A reveals the distribution of trap bands in both 2-ME/DMSO and DMF/NMP devices. PSCs typically exhibit three distinct trap bands (I, II, and III), which correspond to $I_{i}^{-}$ and $I_{i}^{+}$ defects and defects associated with the amorphous regions near the bottom interface (*41*). The results indicate a reduction in the overall trap density in the DMF/NMP devices compared to the 2-ME/DMSO case, with a particularly notable decrease in the density of $I_{i}^{+}$ defects in the depth range of 0.20 to 0.33 eV. The DLCP results at 10 kHz shown in Fig. 4B further corroborate this finding, demonstrating a decrease in overall trap density and a reduction in $I_{i}^{+}$ density. The reduction in $I_{i}^{+}$ density is accompanied by a decrease in the amorphous regions and a reduction in voids at the bottom interface, suggesting a marked improvement in the crystallinity of the perovskite film at its bottom. This finding is consistent with the results obtained from cross-sectional SEM analysis.

We conducted electroluminescence (EL) mapping of complete PSCs to further investigate the micro-uniformity of perovskite films coated using two distinct solvent systems. EL mapping enables the characterization of solar cells in a near-operational state, allowing for quantitative measurement of the luminescent flux of the entire device. This technique has been found to be unaffected by various artifacts that may arise during analysis in a confocal configuration (*42*). A schematic of our EL mapping setup is illustrated in fig. S22. A forward bias voltage is applied to stimulate luminescence, and the injected current density equals the *J*_SC_ under one sun illuminance. Each EL detection area measures 100 μm by 100 μm, with a pixel resolution of 200 nm. The EL mapping provides insights into both the EL intensity and response time. As shown in Fig. 4C, PSCs fabricated from the DMF/NMP precursor solution exhibit much faster EL rise and reach saturation at a higher EL intensity compared to those from the 2-ME/DMSO precursor solution. The rapid EL rise indicates a reduction in interfacial traps, which could adversely affect charge injection and radiative recombination. Conversely, the high EL intensity correlates with suppressed nonradiative recombination, supporting the observation that the corresponding PSCs exhibited the lowest voltage loss. Furthermore, we measured additional randomly selected areas (25 per group) from both sets of PSCs and plotted their average EL response times in Fig. 4D. Notably, PSCs derived from the DMF/NMP precursor solution demonstrate faster EL response times and a narrower distribution compared to the 2-ME/DMSO group, highlighting the intrinsic advantages of the DMF/NMP precursor solution in enhancing device quality. In addition, as illustrated in the EL images (Fig. 4, E and F), the DMF/NMP-based PSCs exhibit superior luminescence uniformity, whereas the 2-ME/DMSO–based cells display poorer uniformity due to the presence of numerous dispersed luminescent centers. These results confirm the advantages of the DMF/NMP ink in achieving uniform crystallization of perovskite films compared to the 2-ME/DMSO ink.

**Fig. 5.**
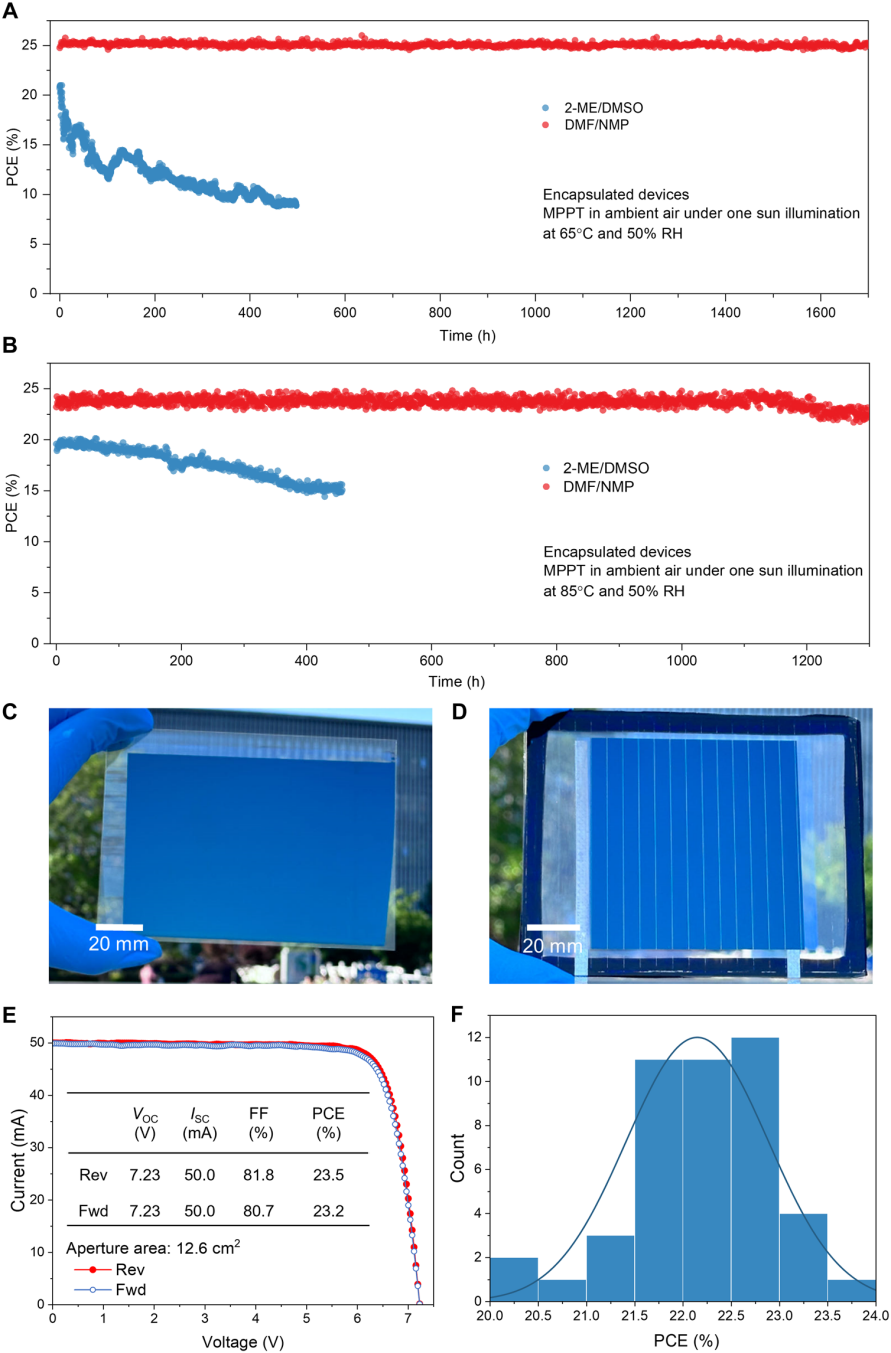
Stability and scalability. (**A**) MPP tracking stability test results of the encapsulated devices soaked under one sun illumination at 65°C in air (50% RH). The initial PCEs of the 2-ME/DMSO and DMF/NMP cells are 20.7 and 25.3%, respectively. (**B**) MPP tracking stability test results of the encapsulated devices soaked under one sun illumination at 85°C in air (50% RH). The initial PCEs of the 2-ME/DMSO and DMF/NMP cells are 19.6 and 23.2%, respectively. Note that atomic layer–deposited SnO*_x_* was used to replace BCP in these devices. (**C**) Photograph of a blade-coated Cs_0.1_FA_0.9_PbI_3_ perovskite film on a large ITO substrate, covering an area of ~130 cm^2^. (**D**) Photograph showcasing an encapsulated minimodule with an aperture area of 63.2 cm^2^. (**E**) *I*-*V* (current-voltage relation) curves of the champion DMF/NMP minimodule (aperture area: 12.6 cm^2^) that has six sub-cells connected in series. (**F**) Efficiency distribution of 45 DMF/NMP perovskite mini-modules.

To evaluate the operational stability of solar cells, we soaked the encapsulated devices in ambient air at 65°C and 50% humidity following the ISOS-L-2 standard protocol and monitored their PCE change under simulated one sunlight illuminance of 100 mW cm^−2^ (Fig. 5A). The DMF/NMP group retained 99% of its initial efficiency after 1700-hour continuous light soaking at the maximum power point tracking (MPPT), whereas the reference cell had degraded to only 43% of its initial efficiency after 500 hours. We also measured the operational stability of three more DMF/NMP devices, and all were able to maintain 98% of their initial PCEs after 1700 hours of MPP tracking (fig. S23), confirming the high stability of DMF/NMP cells. We further measured the device stability at 85°C. To fully evaluate the stability of perovskite layers, we used atomic layer–deposited SnO*_x_* as a protection layer to replace thermally unstable BCP. As shown in Fig. 5B, the device processed from DMF/NMP solution exhibits much better stability at 85°C MPPT tracking condition, and 96.9% of initial PCE was retained after 1300-hour light soaking, while the control device from 2-ME/DMSO ink lost 23% of its PCE after 450 hours.

After validating the performance of small devices using the optimized DMF/NMP solution, we used the same blade coating method to deposit large-area films on ITO substrates. The resulting perovskite film and mini-module exhibited smooth and uniform surfaces (Fig. 5, C and D). Concurrently, we fabricated perovskite solar modules with aperture areas from 10 to 60 cm^2^ and assessed their performance. The sub–cell width was 5.8 mm, achieving a high geometry FF (GFF) of 96.1% and a narrow dead zone width of 225 μm (fig. S24). As shown in Fig. 5E, the perovskite mini-modules reached a maximum PCE of 23.5% from current density-voltage (*J-V*) reverse scanning (with an aperture area of 12.6 cm^2^), a *V*_OC_ of 7.23 V (for 6 sub-cells, yielding a *V*_OC_ of 1.20 V per sub-cell), a short-circuit current (*I*_SC_) of 50.0 mA, and an FF of 0.818. This corresponds to a high active-area efficiency of 24.5% considering the GFF of 96.1%. The champion module also exhibited minimal hysteresis between forward and reverse scans (23.2 and 23.5%, respectively). Furthermore, one of our DMF/NMP mini-modules has been certified by an independent photovoltaic calibration and measurement laboratory, confirming a stabilized aperture PCE of 22.84% (fig. S25). The perovskite modules also showed good reproducibility (Fig. 5F and table S6), where more than 90% of 45 perovskite modules can realize PCEs of more than 21.0%.

## DISCUSSION

In summary, we systematically investigated the intrinsic stability of Cs/FA perovskite inks and unveiled that the solvent coordination-dispersion equilibrium greatly affects ink stability. By modulating solvents with differing coordination strength and solubility properties, we found that DMF/NMP-based perovskite ink exhibited excellent storage stability and extended shelf life. Perovskite layers processed from the DMF/NMP ink exhibited high uniformity and reduced density of trap states, leading to an improved PCEs of 23.5% for air-processed perovskite mini-modules. The DMF/NMP-based PSCs also demonstrated improved operational stability according to the ISOS-L-2 standard protocol.

## MATERIALS AND METHODS

### Materials

PTAA [average *M*_n_ (number-average molecular weight) of 7000 to 10,000], aluminum oxide (Al_2_O_3_) nanoparticles (<50 nm, 20 weight % in isopropanol), PbI_2_ (99.999%), BCP, 2-ME, DMSO, DMF, NMP, LP, benzylhydrazine hydrochloride (BHC), toluene, lithium fluoride (LiF), and toluene were purchased from Merck and used without further purification. FAI, formamidinium chloride (FACl), p-F-PEAI, and *n*-dodecylammonium iodide were purchased from GreatCell Solar. CsI was purchased from Advanced Election Technology Co. Ltd. All chemicals without any notes were commercially available and used without further purification.

### Solar cell fabrication

All fabrication steps—including perovskite solution preparation, PTAA coating, perovskite deposition, thermal annealing, laser scribing, electrode deposition, encapsulation, and measurements—were carried out under ambient conditions (25°C and 30% RH). The prepatterned ITO glass substrates (12 Ω sq^−1^) were first cleaned using ultrasonication with soap, deionized water, and isopropyl alcohol, followed by a 15-min UV-ozone treatment before use. The PTAA and perovskite layers were deposited using blade coating at room temperature inside a fume hood. A PTAA solution with a concentration of 3.3 mg ml^−1^ in toluene and an Al_2_O_3_ nanoparticle solution (10 μl ml^−1^ in isopropanol) were sequentially blade-coated onto ITO glass substrates at a speed of 20 mm s^−1^ using a coating gap of 150 μm, followed by a thermal annealing process at 100°C for 10 min. For the 2-ME/DMSO precursor solution, 1.1 M Cs_0.1_FA_0.9_PbI_3_ solution was prepared by directly mixing A-ink (2.2 M FAI and PbI_2_ in 2-ME) with B-ink (2.0 M CsI and PbI_2_ in DMSO) and then diluting with 2-ME solvent before blade coating. A 0.23% (v/v) FAH_2_PO_3_, *n*-dodecylammonium iodide (0.83 mg ml^−1^), FACl (2.3 mg ml^−1^), LP (0.27 mg ml^−1^), CsI (0.57 mg ml^−1^), p-F-PEAI (1.40 mg ml^−1^), ZnCl_2_ (0.74 mg ml^−1^), and BHC (0.15 mg ml^−1^) were added into the precursor solutions as additives. The precursor solution was then blade-coated onto ITO glass substrates using a gap of 300 μm and a movement speed of 20 mm s^−1^. The air knife step is carried out immediately after blade coating, with a time interval of less than 1 s. The air knife was operated at a pressure of 206,843 Pa during the coating process. Following this, the perovskite films were annealed at 150°C for 5 min in ambient air. For the DMF/NMP precursor solution, a 1.1 M precursor solution of Cs_0.1_FA_0.9_PbI_3_ was prepared in a solvent mixture of DMF and NMP at a volume ratio of 5.2:1. Additional components were incorporated into the solution as additives, including *n*-dodecylammonium iodide (0.83 mg ml^−1^), LP (0.27 mg ml^−1^), CsI (0.57 mg ml^−1^), p-F-PEAI (1.40 mg ml^−1^), ZnCl_2_ (0.74 mg ml^−1^), and BHC (0.15 mg ml^−1^). The precursor solution was then blade-coated onto ITO glass substrates using a gap of 300 μm and a movement speed of 20 mm s^−1^. The air knife step is carried out immediately after blade coating, with a time interval of less than 1 s. The air knife was operated at a pressure of 30 psi during the coating process. Following this, the perovskite films were annealed at 120°C for 30 min in ambient air. The fabrication of the solar cells was completed by thermally evaporating LiF (0.6 nm at 0.1 Å s^−1^), C_60_ (30 nm at 0.25 Å s^−1^), BCP (6 nm at 0.1 Å s^−1^), and 100-nm copper (0.5 Å s^−1^). For the stability of the solar cells tested at 85°C, atomic layer–deposited SnO*_x_* was used to replace BCP. The deposition process was conducted at 100°C under a high-purity nitrogen (99.999%) environment, which served as both carrier and purge gas. Gas flows for the chamber and process were maintained at 30 standard cubic centimeter per minute (sccm) each. A 15-nm-thick SnO*_x_* film was deposited via 140 cycles.

### Module fabrication

The coating of large-area perovskite films followed the same method as the PSCs. In the fabrication of the mini-modules, the laser processing parameters for P1 were meticulously optimized using a power of 0.55 W, *Q* frequency of 80 kHz, and a scanning speed of 500 mm/s, which yielded a line width of 20 μm for the P1 scribing. Subsequent laser scribing for P2 was conducted at a power of 1.75 W, *Q* frequency of 280 kHz, and a scanning speed of 500 mm/s, while P3 scribing was performed at a power of 1.5 W, *Q* frequency of 260 kHz, and a scanning speed of 500 mm/s. Each sub-cell was designed with a nominal width of 5.8 mm, with the final measured widths for P2 and P3 being ~134 and 47 μm, respectively. The laser scribing process was conducted using a Keyence laser marker (MD-U1000C, 355 nm), resulting in a nonworking area width of ~225 μm, which corresponds to a GFF of 96.1%. To improve the light harvesting of the perovskite solar modules, a polydimethylsiloxane layer, fabricated via a soft lithography technique, was applied as an antireflection coating to the front surface of the glass substrate. The GFF of the mini-modules was determined through analysis of height-scanning distance curves acquired using a Keyence 3D Surface Profiler (VK-X3050).

### Device characterization

The *J-V* characteristics of solar devices were measured using a 3A Class solar simulator (BG-LED3A-100S, Class AAA Solar Simulator), and the power of the simulated light was calibrated to 100 mW cm^−2^ by a silicon reference cell (Newport 91150 V). All devices were measured using a Keithley 2400 source meter with a scan rate of 0.1 V s^−1^ in the air at room temperature, and the delay time was 10 ms. There was no preconditioning before measurement. A thin mosmite-type film was applied to the front surface of glass substrate as an antireflection coating during the measurement of the PSCs. A metal mask with an aperture (6.84 mm^2^) aligned with the solar cell active area was used for measurements. The PSCs were encapsulated with cover glass sealed by an epoxy encapsulant (Gorilla 2-part epoxy 4200130) on the back. After curing, the stability of encapsulated cells was monitored with a 91 PVKSOLAR MSCLT-1 automatic MPP tracker, and all cells kept working at MPP conditions under simulated AM 1.5G one sun illumination (100 mW cm^−2^) in air (50% RH). The solar cells were mounted on a hot plate, and the temperature of the cells maintained at 65° or 85°C measured by a hand-held thermometer. The stabilized PCEs were recorded every 10 s. The EQE spectra were obtained with a Zolix SCS600 solar cell quantum efficiency measurement system with a standard Si cell. The XRD patterns were acquired with a Bruker D8 ADVANCE x-ray diffractometer. The instrument used for the SEM characterization is TESCAN MIRA3 LMH. Atomic force microscopy height images were obtained with a Bruker Icon atomic force microscope. TOF-SIMS depth profiling was performed using an IONTOF TOF-SIMS 5 instrument (IONTOF Co., Germany). tDOS and DLCP spectra were obtained with an Agilent E4980A precision LC meter. DLS experiments were conducted by a DLS particle size analyzer (BI-200SM, Brookhaven Instruments Corporation) at 25°C. In situ attenuated total reflection (ATR) FTIR spectra were collected using a Bruker Tensor II spectrometer equipped with a platinum ATR accessory (diamond crystal, single reflection), a DigiTect digital detector, and an integrated RockSolid interferometer. Steady-state PL spectra were acquired on a HORIBA FL-3 fluorescence spectrophotometer at room temperature. The excitation wavelengths were 485 nm with an intensity of ~60 mW cm^−2^. The imaging system of EL mapping was conducted using an inverted microscope (Eclipse Ti-U, Nikon) with an Autolab potentiostat (PGSTAT302N). A forward voltage was applied to stimulate luminescence. The optical images were obtained using a metallurgical microscope with a large stage (NX600, NEXCOPE). The EL light from the cell was captured by a charge-coupled device camera (Stingray, Allied Vision Technologies) through a 40× objective (numerical aperture = 0.6). A data acquisition card (USB-6281, National Instruments) was used to synchronize the voltage output from the potentiostat and transistor-transistor logic signals from the camera.

### In situ UV-vis absorption spectroscopy measurements

A 100-nm layer of silver was deposited on the backside of ITO glass to serve as a reflective layer for measuring the reflective absorption of the perovskite during the blade coating. Sampling commenced before the blade-coating process, with an interval of 100 ms between samples. A Bobaylight BBZM-III xenon lamp (spectral range: 300 to 1100 nm) was used as the UV-vis light source, while an IDEAOPTICS NOVA 2S detector (detection range: 325 to 1100 nm) was used for measurements.
