# Supplementary material for: Stabilized perovskite ink for scalable coating enables high-efficiency perovskite modules
Source: Sci Adv. 2026 Jan 2;12(1):eaec0915. doi: 10.1126/sciadv.aec0915 (PMC12758550; doi:10.1126/sciadv.aec0915)
Supplement: Supplementary file 1 — Figs. S1 to S25 Tables S1 to S6 [file sciadv.aec0915_sm.pdf]

Science Advances
AAAS

Supplementary Materials for

Stabilized perovskite ink for scalable coating enables high-efficiency perovskite modules

Yangyang Liu *et al.*

Corresponding author: Henry J. Snaith, henry.snaith@physics.ox.ac.uk; Shangshang Chen, schen@nju.edu.cn

Sci. Adv. **12**, eaec0915 (2026)
DOI: 10.1126/sciadv.aec0915

This PDF file includes:

Figs. S1 to S25
Tables S1 to S6

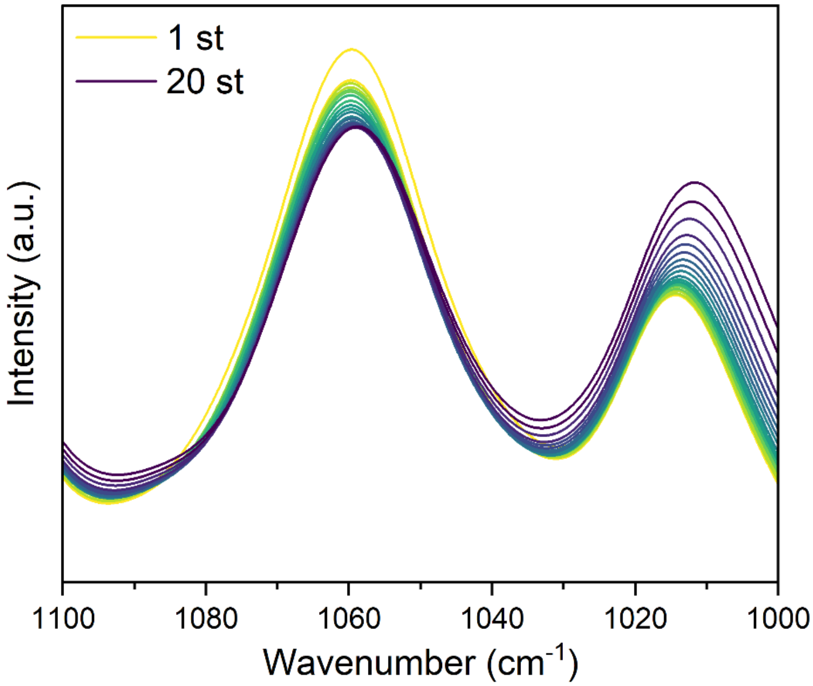

Fig. S1. Partial enlarged in situ FT-IR spectra (1000 to 1100 cm^{-1}) of the 2-ME/DMSO ink.

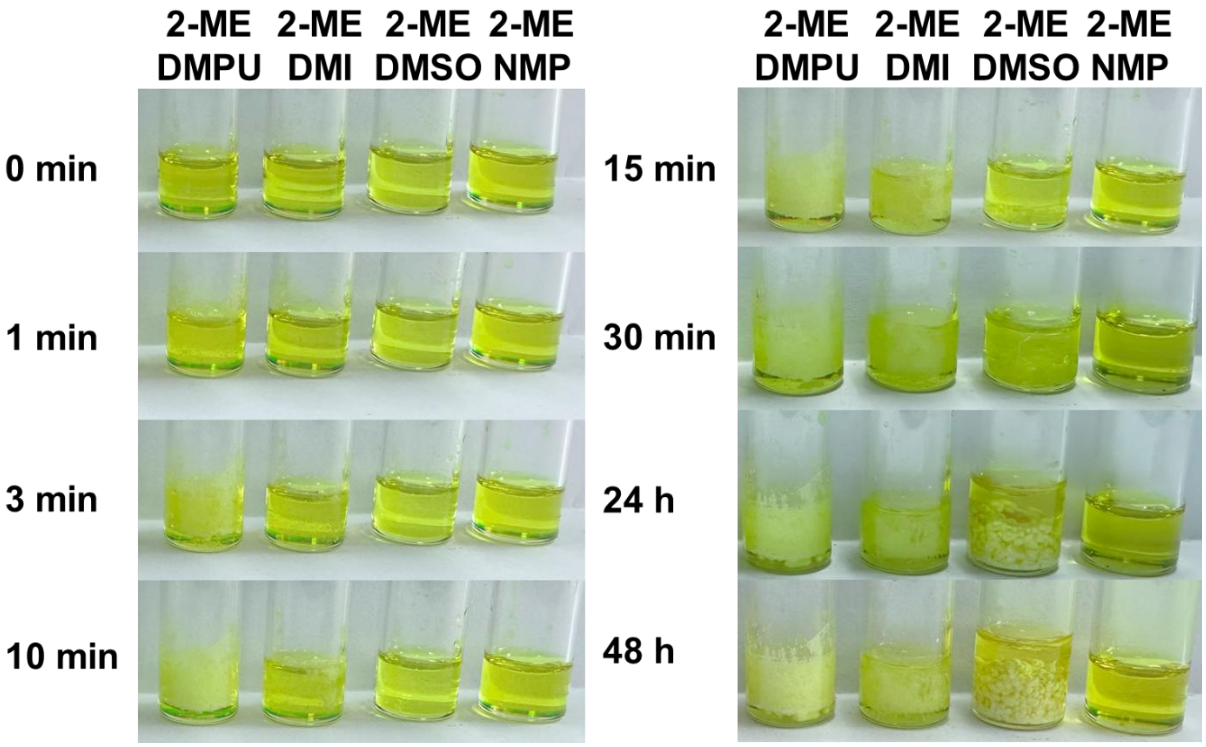

Fig. S2. The photographs of $Cs_{0.1}FA_{0.9}PbI_3$ solutions dissolved in 2-ME and various coordinating solvents stored in air (25°C, 50% RH) for 48 hours.

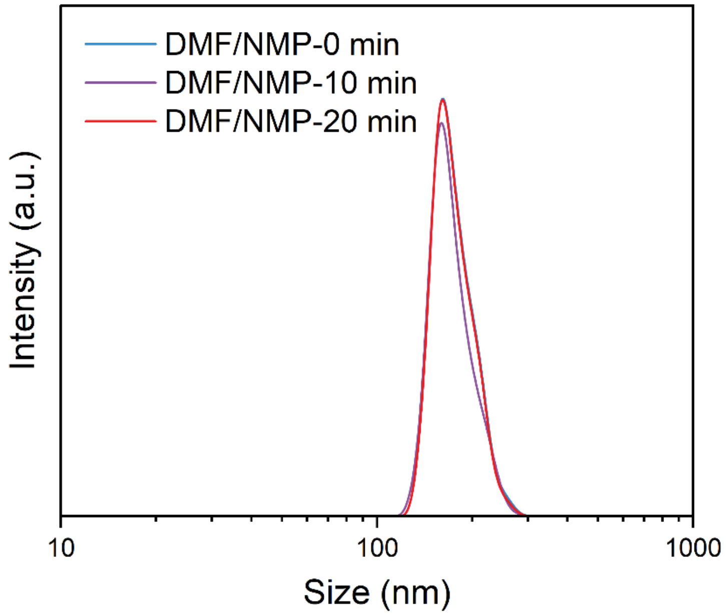

Fig. S3. DLS spectra of the $Cs_{0.1}FA_{0.9}PbI_3$ DMF/NMP ink after different storage durations.

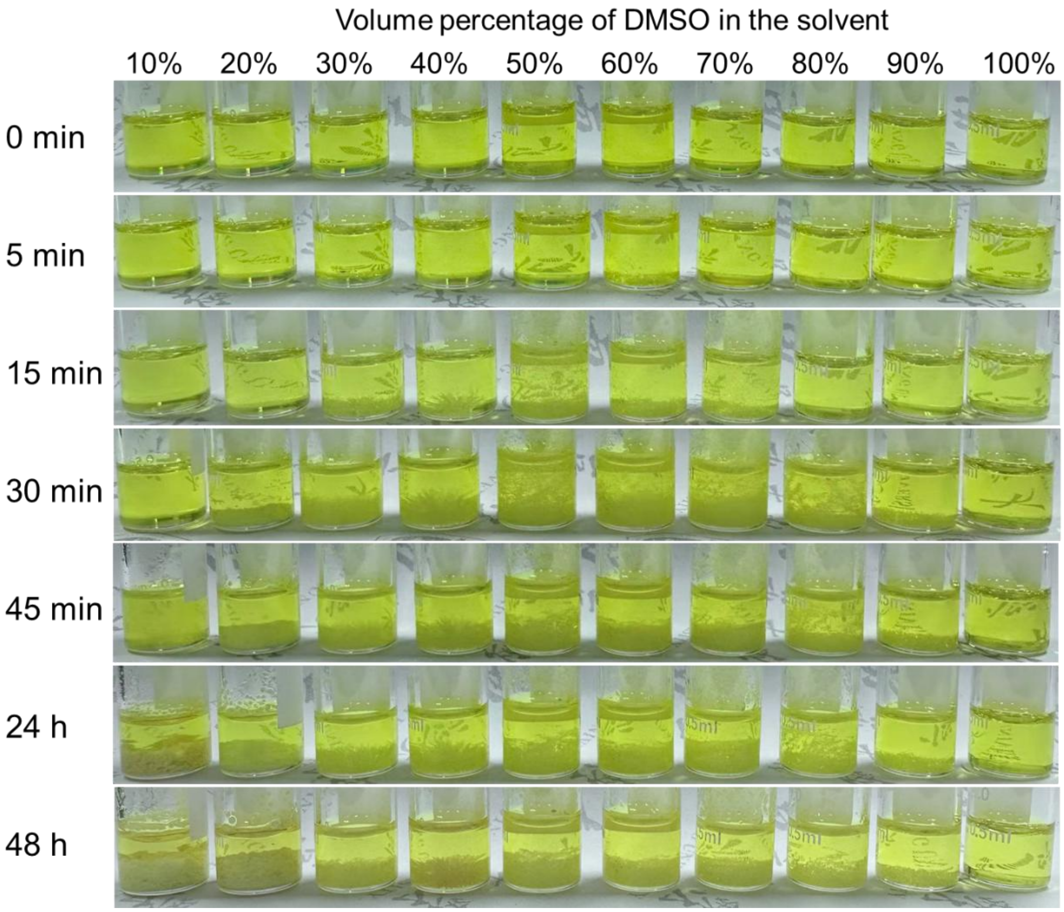

Fig. S4. Storage stability of $Cs_{0.1}FA_{0.9}PbI_3$ precursor solutions with varied 2-ME:DMSO volume ratios (from 9:1 to 0:10), monitored for 48 hours under ambient conditions (25°C, 50% RH).

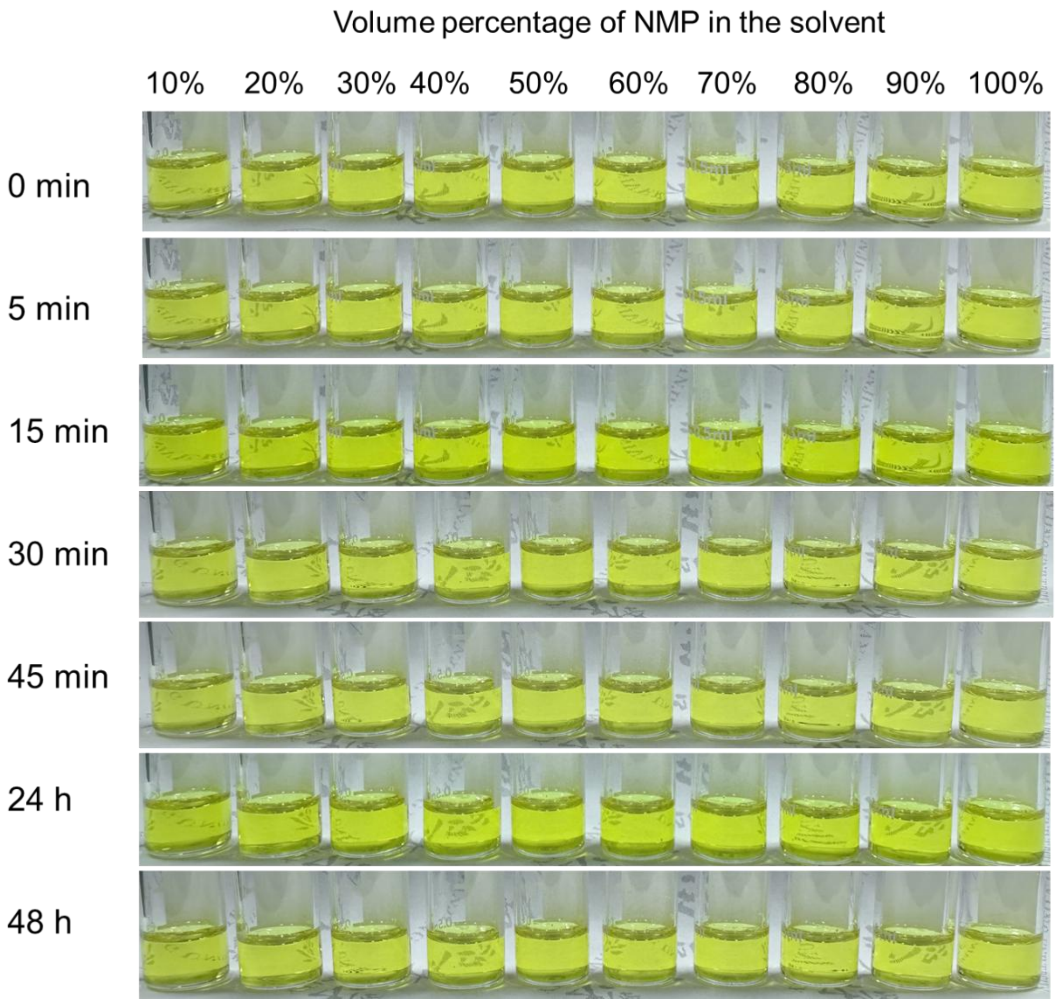

Fig. S5. Storage stability of $Cs_{0.1}FA_{0.9}PbI_3$ precursor solutions with varied DMF:NMP volume ratios (from 9:1 to 0:10), monitored for 48 hours under ambient conditions (25°C, 50% RH).

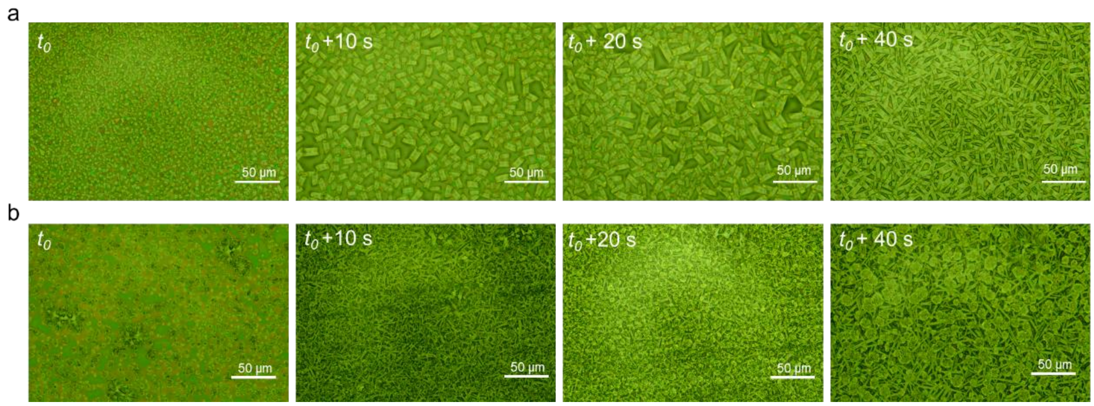

Fig. S6. Optical images of the wet films as a function of time for (a) 2-ME/DMSO and (b) DMF/NMP-based perovskite films.

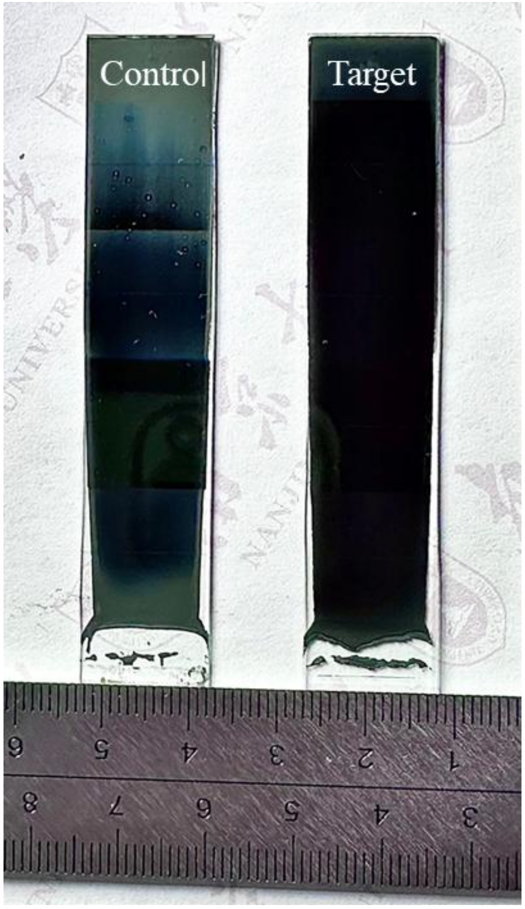

Fig. S7. The photographs of the 2-ME/DMSO (left) and DMF/NMP (right) perovskite films blade-coated on $1.5 \times 9\ cm^2$ ITO/PTAA substrates. The DMF/NMP perovskite film is more uniform than the 2-ME/DMSO film.

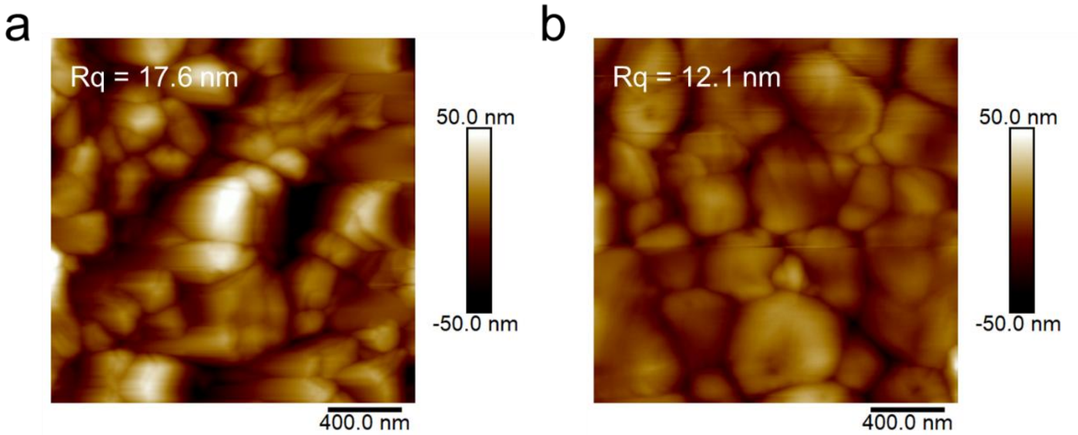

Fig. S8. AFM height images of (a) 2-ME/DMSO and (b) DMF/NMP perovskite films.

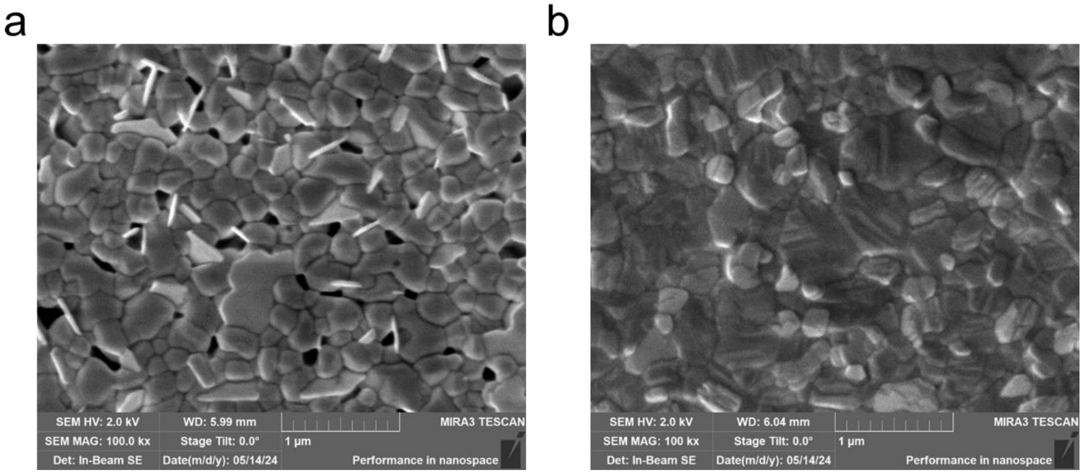

Fig. S9. Top-view SEM images of (a) 2-ME/DMSO and (b) DMF/NMP perovskite films.

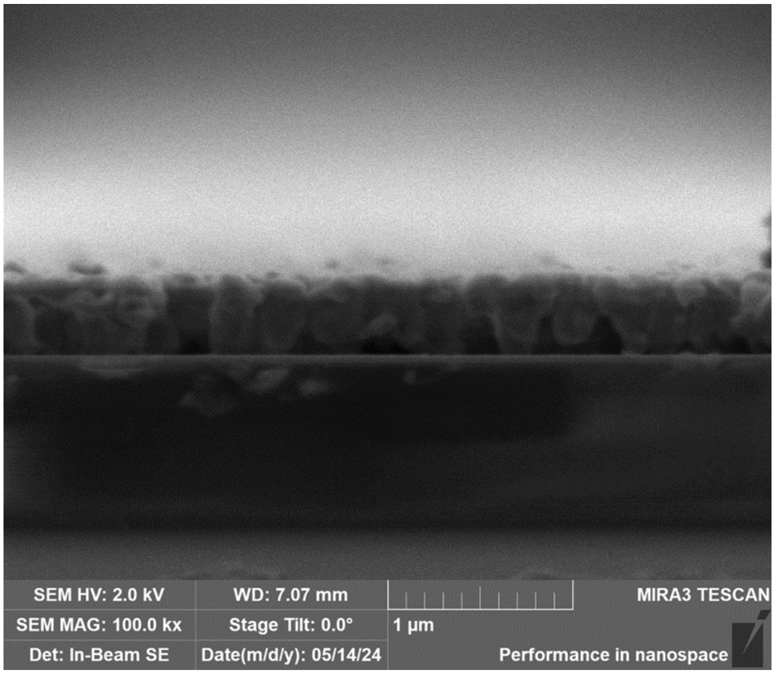

Fig. S10. Cross-sectional SEM image of the 2-ME/DMSO perovskite film.

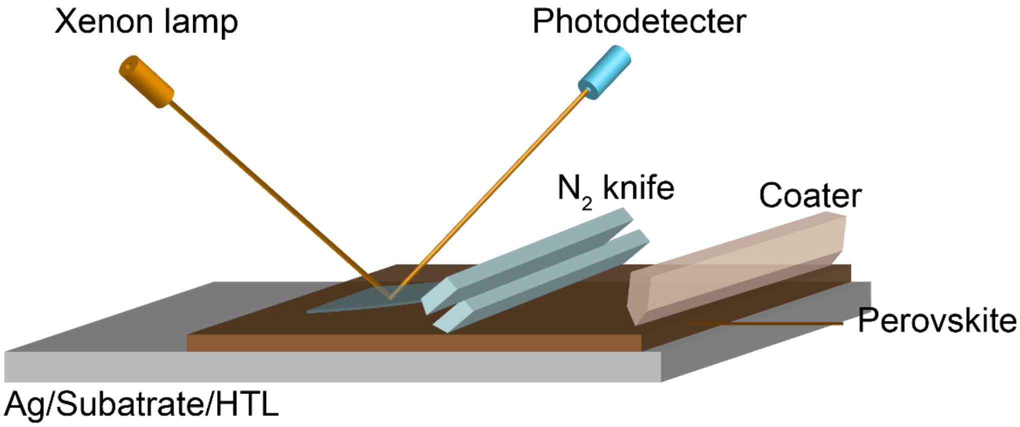

Fig. S11. The setup for in situ UV-vis absorption measurement.

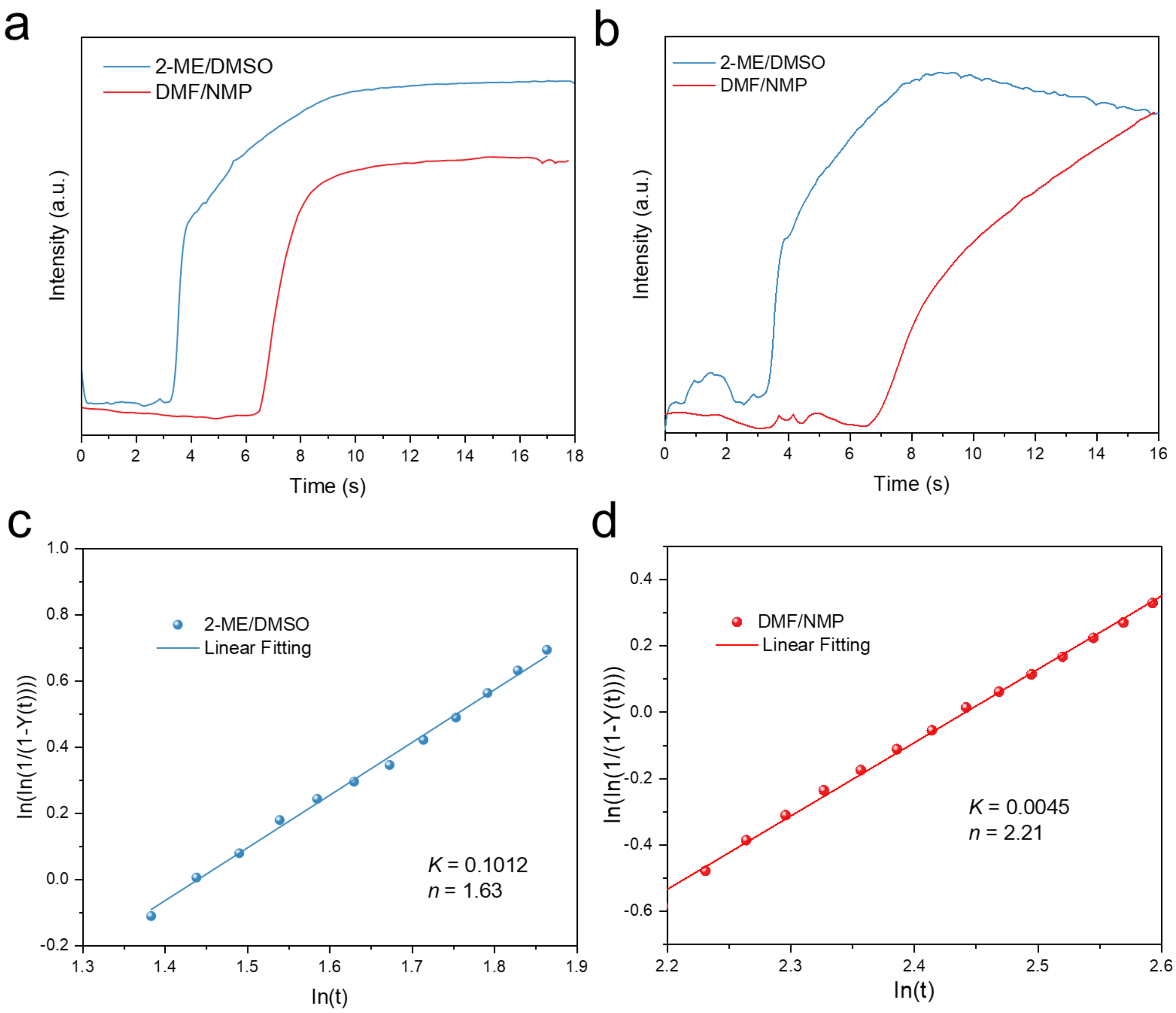

Fig. S12. In situ UV-vis film absorption profiles of the 2-ME/DMSO and DMF/NMP perovskite inks. (a) Absorption intensity evolution at 450 nm at the end of blade coating. (b) Absorption intensity evolution at 720 nm and the cutoff absorption intensity at 760 nm of the wet film at the end of blade coating. (c), (d) Linear fitting for the ln(-ln(1-Y(*t*))) versus ln(*t*) plot of two perovskite films to determine the dimensionality (*n*) and rate constant (*K*).

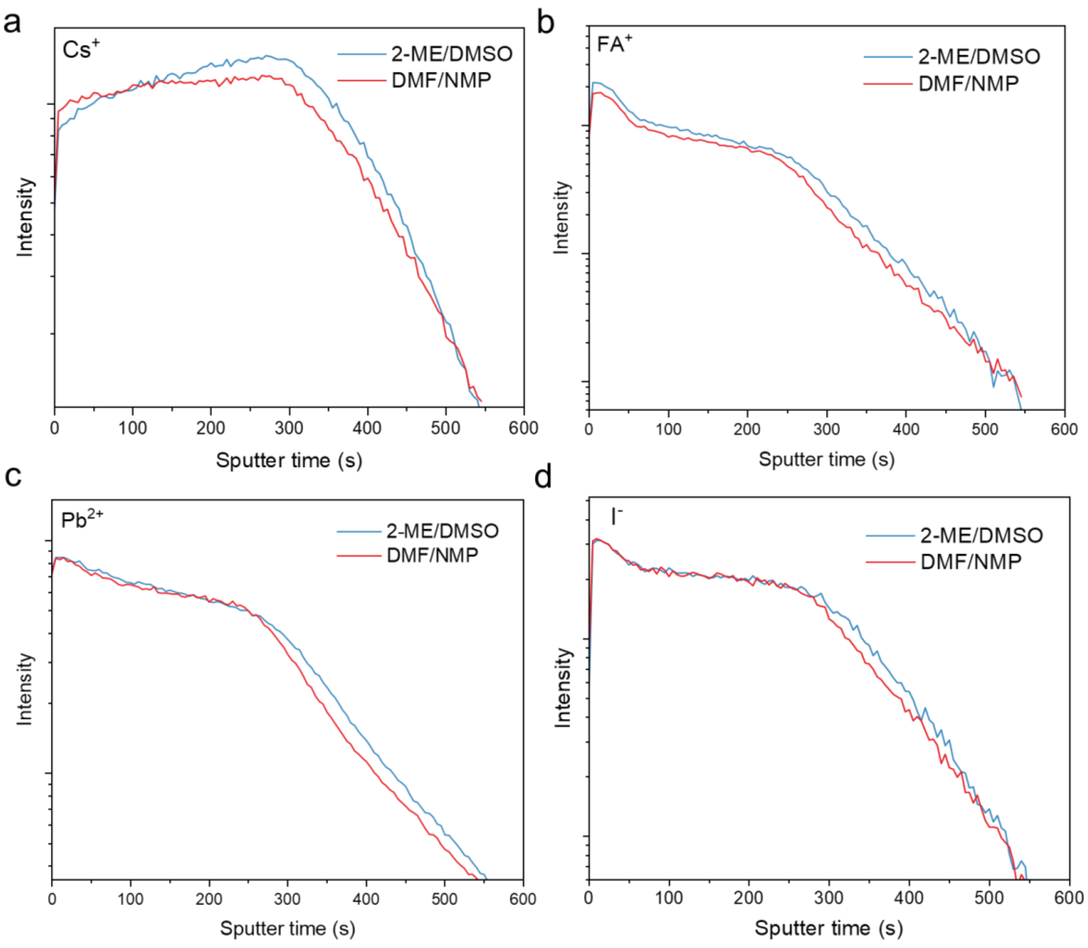

Fig. S13. TOF-SIMS ion distribution profiles of the 2-ME/DMSO and DMF/NMP films (intensity is log-scaled).

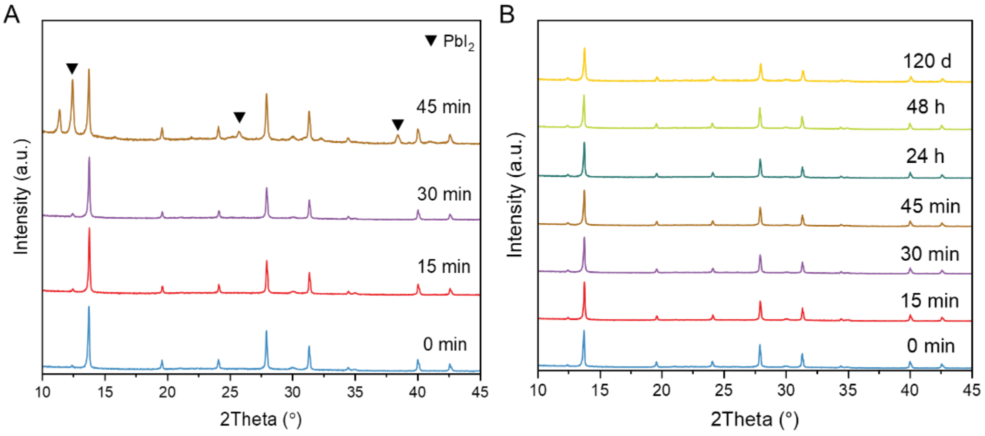

Fig. S14. XRD patterns of the perovskite films processed from (A) 2-ME/DMSO and (B) DMF/NMP inks aged for various durations.

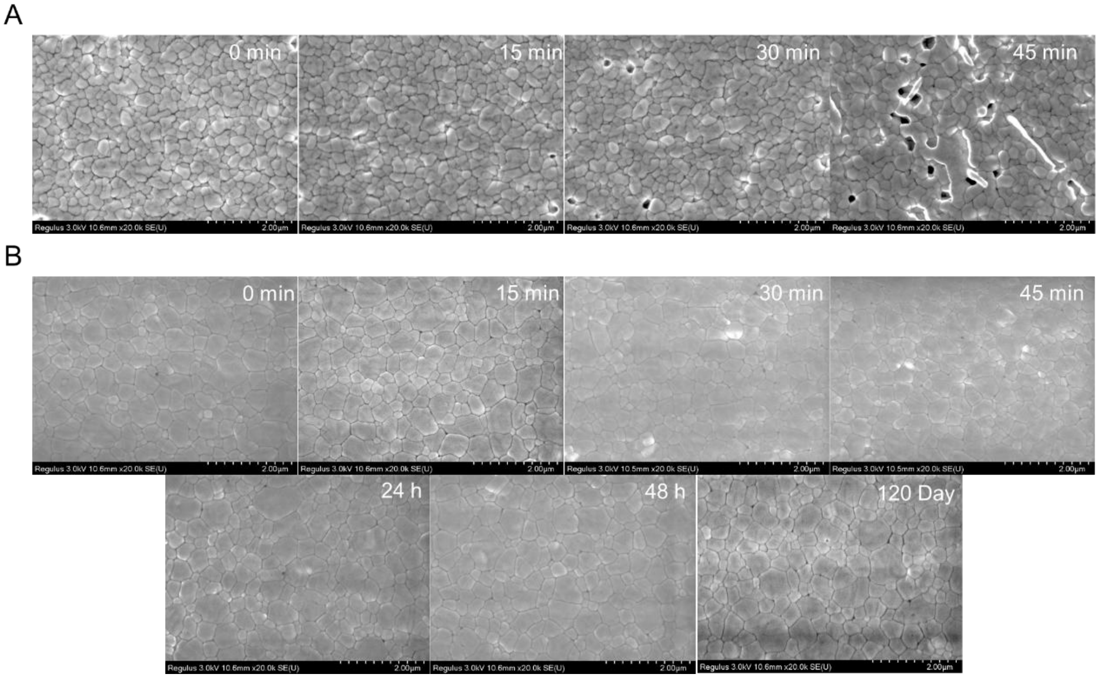

Fig. S15. Top-view SEM images of the perovskite films processed from (A) 2-ME/DMSO and (B) DMF/NMP inks aged for various durations.

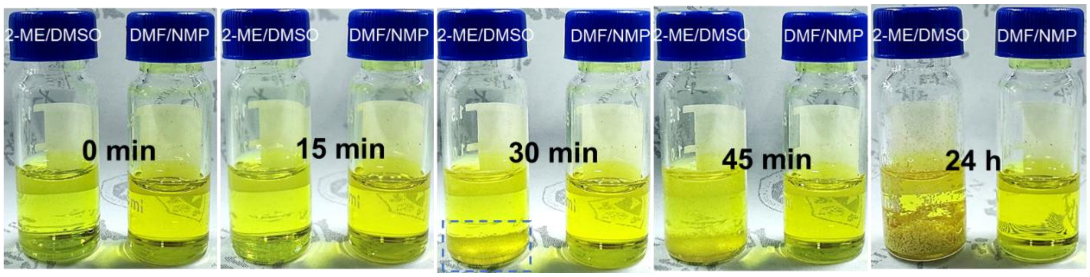

Fig. S16. The photographs of $Cs_{0.1}FA_{0.9}PbI_3$ in 2-ME/DMSO (left) and DMF/NMP (right) solvents stored in air (25°C, 50% RH) for 24 hours. These two solutions contain no additives.

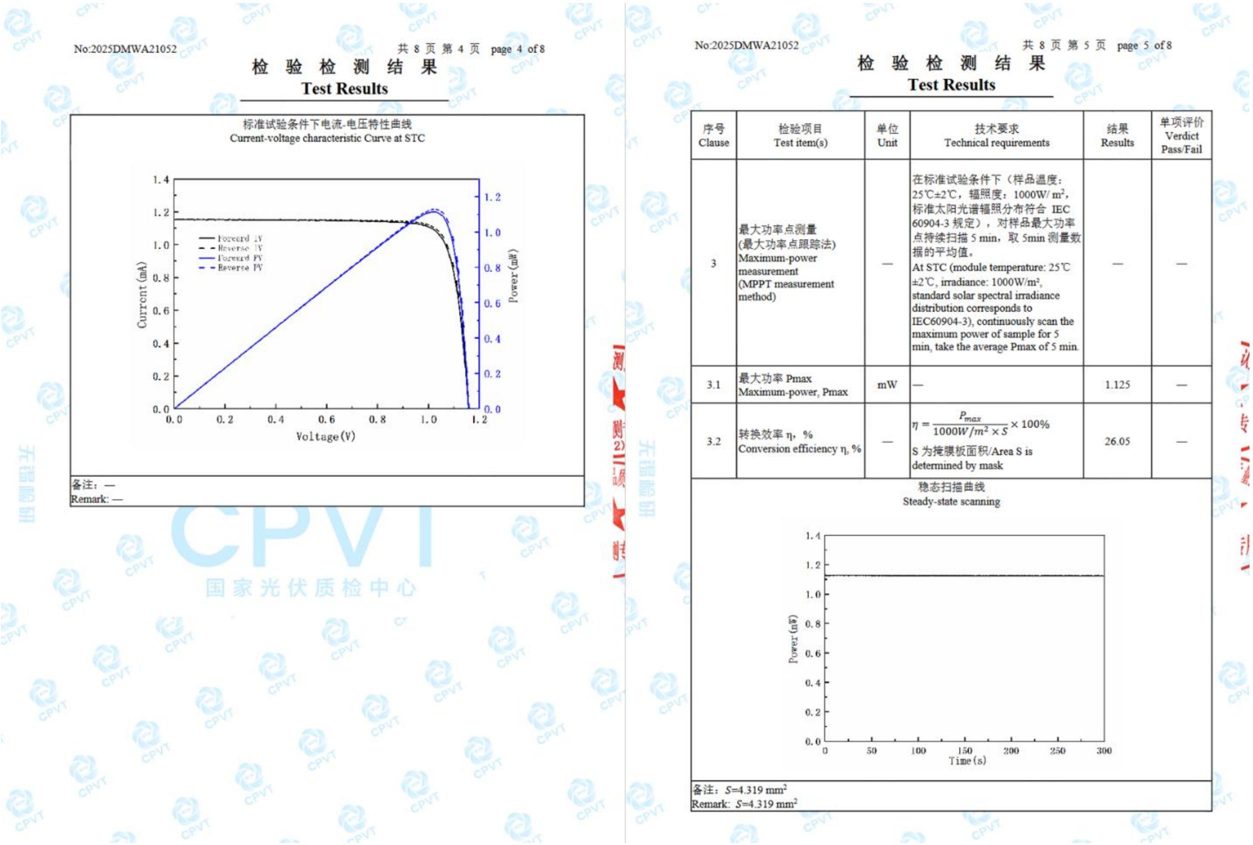

No:2025DMWA21052 共 8 页 第 4 页 page 4 of 8

检 验 检 测 结 果

Test Results

标准试验条件下电流-电压特性曲线

Current-voltage characteristic Curve at STC

备注：—

Remark: —

No:2025DMWA21052 共 8 页 第 5 页 page 5 of 8

检 验 检 测 结 果

Test Results

序号 Clause	检验项目 Test item(s)	单位 Unit	技术要求 Technical requirements	结果 Results	单项评价 Verdict Pass/Fail
3	最大功率点测量 (最大功率点跟踪法) Maximum-power measurement (MPPT measurement method)	—	在标准试验条件下（样品温度：25℃±2℃，辐照度：1000W/ m²，标准太阳光谱辐照分布符合 IEC 60904-3 规定），对样品最大功率点持续扫描 5 min，取 5min 测量数据的平均值。At STC (module temperature: 25℃ ±2℃, irradiance: 1000W/m², standard solar spectral irradiance distribution corresponds to IEC60904-3), continuously scan the maximum power of sample for 5 min, take the average Pmax of 5 min.	—	—
3.1	最大功率 Pmax Maximum-power, Pmax	mW	—	1.125	—
3.2	转换效率 η，% Conversion efficiency η, %	—	$\eta = \frac{P_{max}}{1000W/m^2 \times S} \times 100\%$ S 为掩膜板面积/Area S is determined by mask	26.05	—

稳态扫描曲线

Steady-state scanning

备注：S=4.319 mm²

Remark: S=4.319 mm²

Fig. S17. Certification of photovoltaic performance of the DMF/NMP-based cell (4.319 mm^2) measured by the National Center of Inspection on Solar Photovoltaic Products Quality (CPVT).

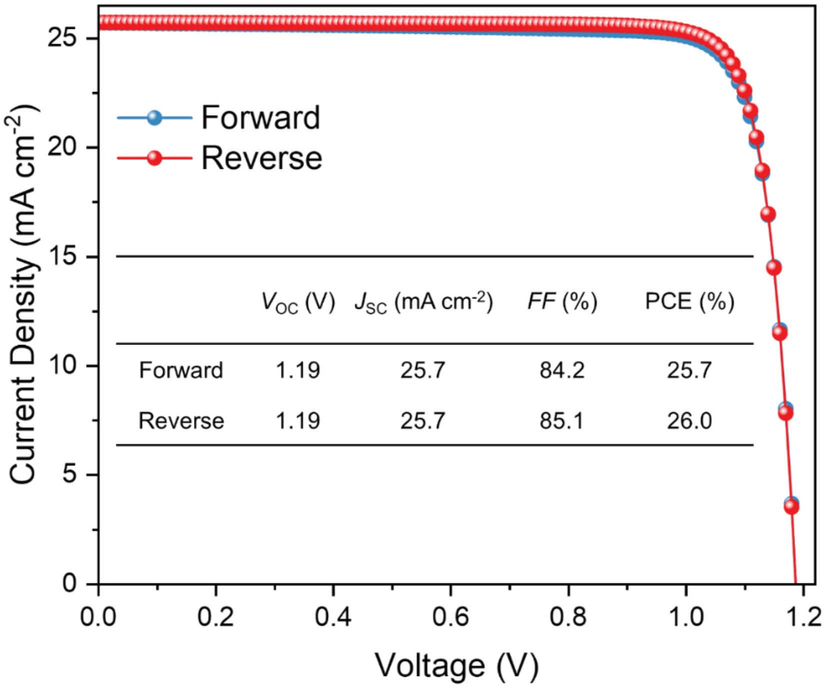

Fig. S18. Reverse and forward *J*-*V* characteristic curves of the DMF/NMP PSCs. Inset shows the device parameters.

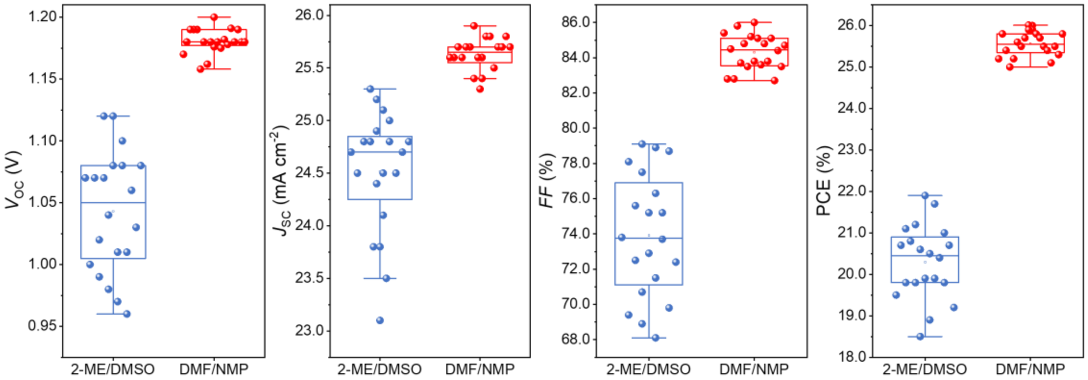

Fig. S19. Statistical analysis of PSC parameters derived from reverse *J*-*V* sweeping based on 2-ME/DMSO and DMF/NMP precursor solutions. Box plot elements are defined as follows: center line: median; hollow square: mean; box limits: upper and lower quartiles; whiskers: 1.5× interquartile range.

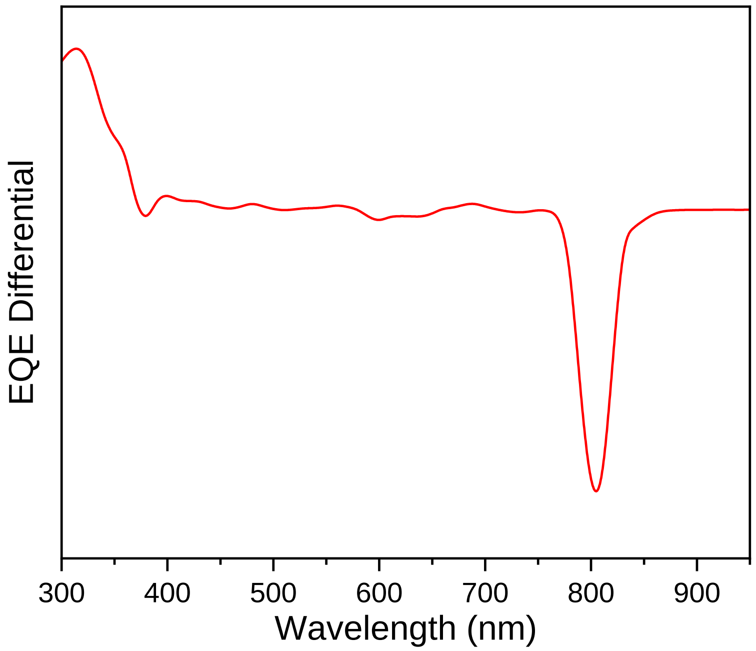

Fig. S20. Differential plot from EQE spectrum of the champion DMF/NMP device.

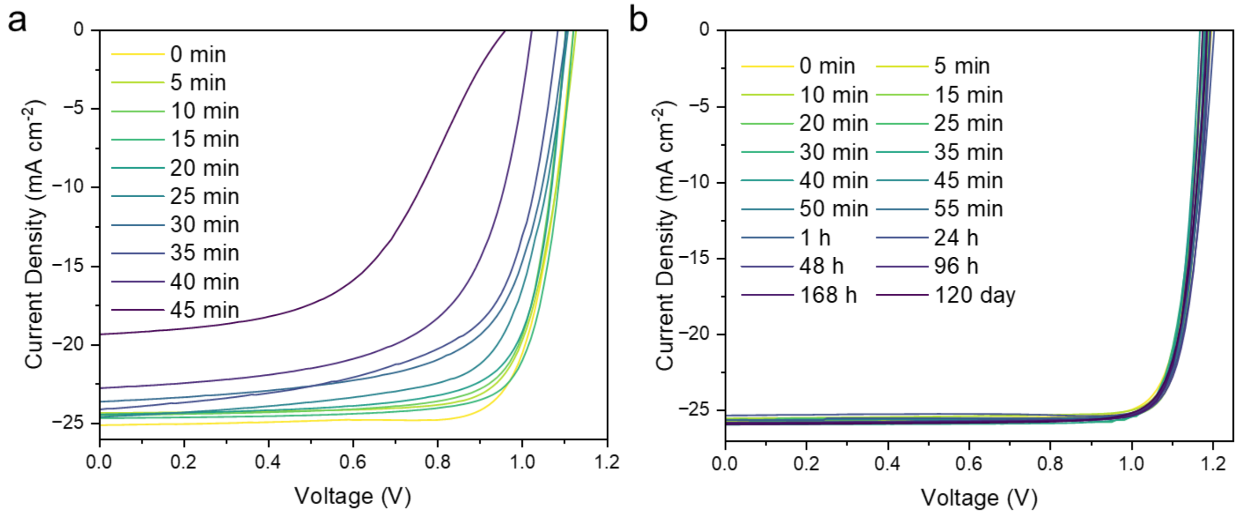

Fig. S21. The reverse *J-V* curves of the PSCs processed from (a) 2-ME/DMSO and (b) DMF/NMP solutions with different aging interval.

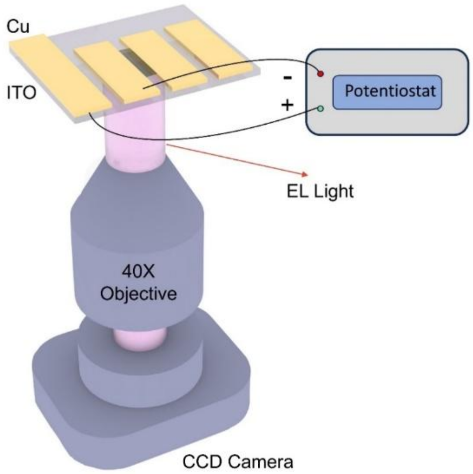

Fig. S22. Schematic illustration of EL mapping characterization setup.

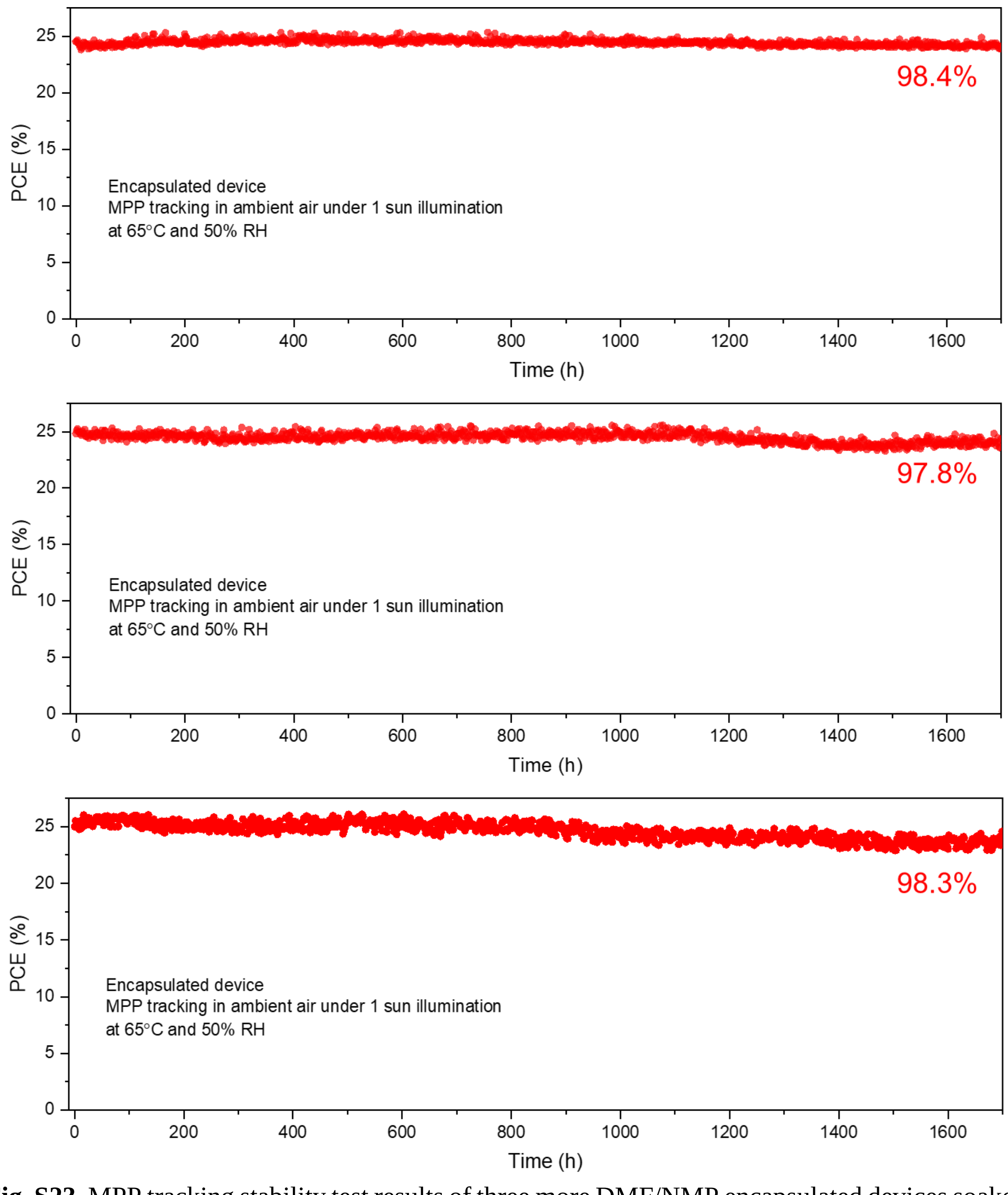

Fig. S23. MPP tracking stability test results of three more DMF/NMP encapsulated devices soaked under one sun illumination at 65°C in air (50% RH).

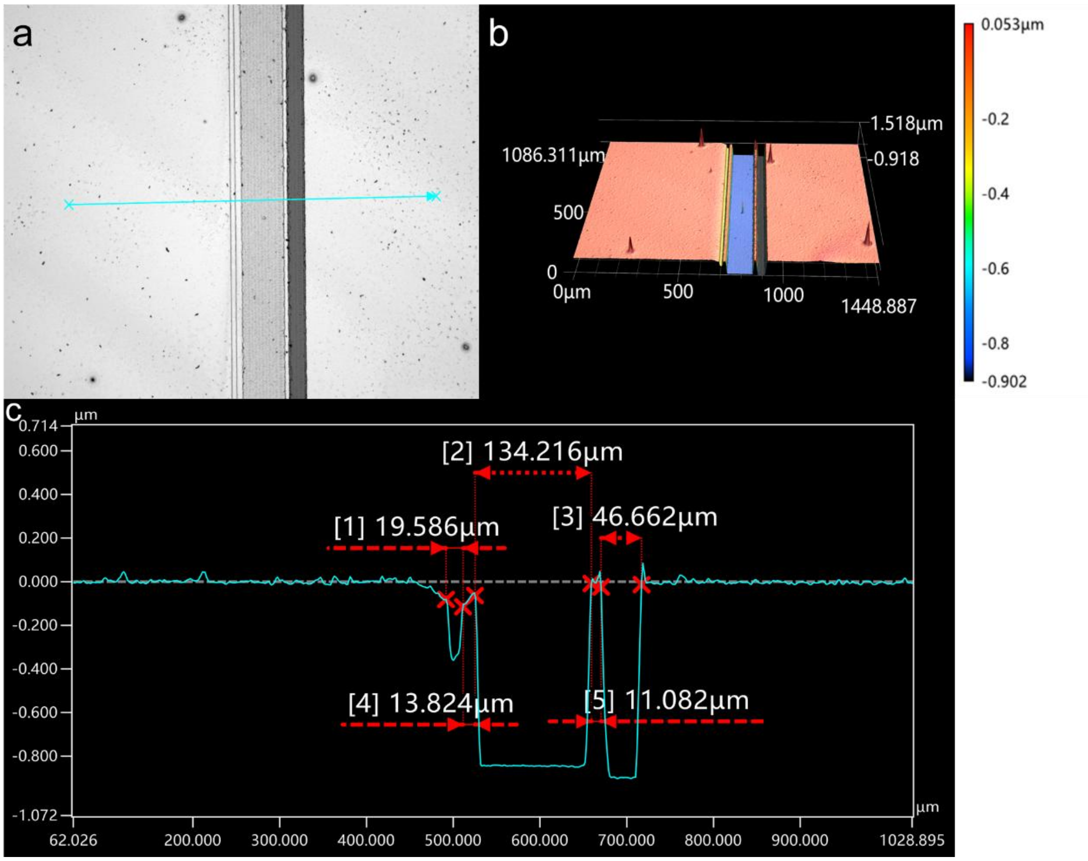

Fig. S24. The geometry of the perovskite solar modules. (a) Optical microscopy image, (b) 3D profile, and (c) surface profiling of laser-scribed module P1, P2, and P3 regions.

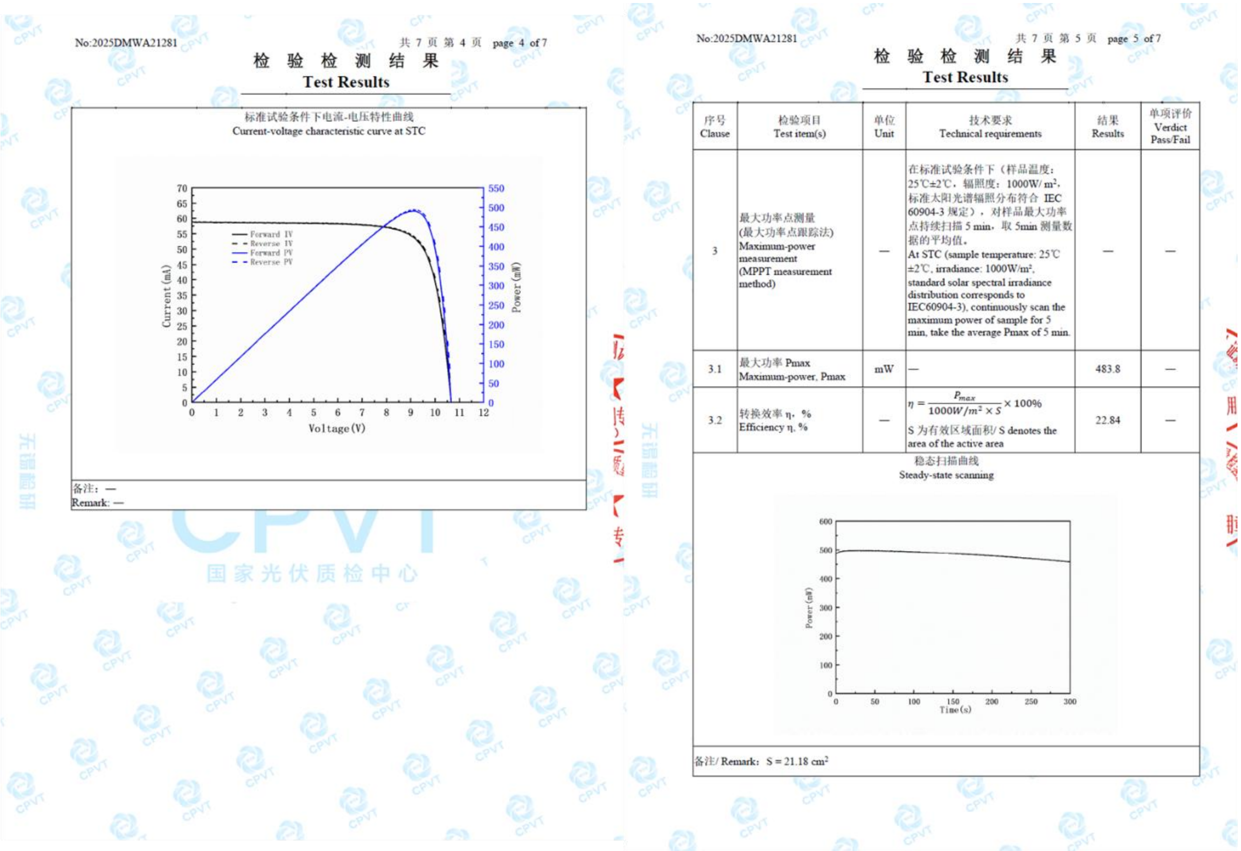

No:2025DMWA21281 共 7 页 第 4 页 page 4 of 7

检 验 检 测 结 果

Test Results

备注：—
Remark: —

No:2025DMWA21281 共 7 页 第 5 页 page 5 of 7

检 验 检 测 结 果

Test Results

序号 Clause	检验项目 Test item(s)	单位 Unit	技术要求 Technical requirements	结果 Results	单项评价 Verdict Pass/Fail
3	最大功率点测量 (最大功率点跟踪法) Maximum-power measurement (MPPT measurement method)	—	在标准试验条件下（样品温度：25℃±2℃，辐照度：1000W/ m²，标准太阳光谱辐照分布符合 IEC 60904-3 规定），对样品最大功率点持续扫描 5 min，取 5min 测量数据的平均值。 At STC (sample temperature: 25℃ ±2℃, irradiance: 1000W/m², standard solar spectral irradiance distribution corresponds to IEC60904-3), continuously scan the maximum power of sample for 5 min, take the average Pmax of 5 min.	—	—
3.1	最大功率 Pmax Maximum-power, Pmax	mW	—	483.8	—
3.2	转换效率 η，% Efficiency η, %	—	$\eta = \frac{P_{max}}{1000W/m^2 \times S} \times 100\%$ S 为有效区域面积/ S denotes the area of the active area	22.84	—

稳态扫描曲线
Steady-state scanning

备注/ Remark: S = 21.18 cm²

Fig. S25. Certification of photovoltaic performance of the DMF/NMP-based mini-module (21.18 cm^2 aperture area) measured by the National Center of Inspection on Solar Photovoltaic Products Quality (CPVT).

Table S1. Processing details of the additives used in the perovskite inks.

Additive	Concentration (mg ml^{-1})	Function	Reference
L-α-phosphatidylcholine	0.27	Facilitate the ink spreading and tune the fluid drying dynamics	*Nat. Energy* 2018, 3, 560.
$C_{12}H_{25}NH_3I$	0.83	Passivate the interfacial defects	*Adv. Energy Mater.* 2019, 9, 1902740.
$ZnCl_2$	0.74	Doping perovskite films	*Nat. Commun.* 2024, 15, 1355.
BHC	0.15	Prohibit the oxidation of I^-	*Sci. Adv.* 2021, 7, eabe8130.
FAH_2PO_3	2.3	Tune crystallization	*Energy Environ. Sci.* 2016, 9, 867.
FACl	2.3	Optimize grain size and film morphology	*Nat. Energy* 2021, 6, 633.
p-F-PEAI	1.4	Surface defect passivation	*Sci. Adv.* 2021, 7, eabj7930.
CsI	0.57	Compensates iodide vacancies	*Nat. Energy* 2021, 6, 633.

Table S2. Photovoltaic performance of the air-processed PSCs reported recently.

Device structures	Fabrication method	V_{OC} (V)	J_{SC} (mA cm^{-2})	*FF* (%)	PCE (%)	References
n-i-p	Spin coating	1.17	25.34	81.36	24.10	*Science* 2021, 371, 1359
n-i-p	Slot-die coating	1.178	24.03	82.5	23.35	*Science* 2021, 372, 1327-1332
p-i-n	Blade coating	1.17	23.9	83.6	23.2	*Sci. Adv.* 2021, 7, 10
p-i-n	Blade coating	1.17	24.1	84.2	23.8	*Science* 2021, 373, 902
n-i-p	Spin coating	1.16	24.8	83.2	23.91	*Joule* 2022, 6, 2203
n-i-p	Spin coating	1.17	25.52	80.6	24.13	*Nat.Commun.* 2022 13, 89
p-i-n	Blade coating	1.17	25.5	82.5	24.6	*Science* 2023, 380, 823
n-i-p	Spin coating	1.174	25.90	83.26	25.32	*Nat. Energy* 2023, 8, 1158
n-i-p	Spin coating	1.20	25.73	81.82	25.18	*Adv. Mater.* 2024, 2411982
p-i-n	Spin coating	1.185	25.36	84.6	25.42	*Nat. Energy* 2024, 9, 536
n-i-p	Spin coating	1.191	24.85	84.9	25.13	*Adv. Mater.* 2024, 36, 2411721
p-i-n	Blade coating	1.19	25.9	81.4	25.1	*Adv. Mater.* 2024, 36, 2402785
n-i-p	Spin coating	1.179	25.2	82.9	24.6	*Science* 2024, 385, 161
n-i-p	Spin coating	1.18	25.9	84.2	25.71	*ACS Energy Lett.* 2024, 9, 7, 3418
**p-i-n**	**Blade coating**	**1.19**	**25.7**	**85.1**	**26.0**	**This work**

Table S3. Summary of photovoltaic parameters for the PSCs prepared from the 2-ME/DMSO precursor solution at different time intervals.

Time (min)	V_{OC} (V)	J_{SC} (mA cm^{-2})	*FF* (%)	PCE (%)
0	1.11	25.1	77.9	21.8
5	1.12	24.3	76.8	21.1
10	1.10	24.5	76.9	20.7
15	1.12	24.7	78.5	21.7
20	1.10	24.4	75.4	20.3
25	1.07	25.0	72.4	19.3
30	1.11	22.9	67.2	17.1
35	1.08	24.1	64.1	16.7
40	1.10	21.9	65.9	15.9
45	1.07	21.41	57.3	13.1

Table S4. Summary of photovoltaic parameters for the PSCs prepared from the DMF/NMP precursor solution at different time intervals.

**Time**	V_{OC} **(V)**	J_{SC} **(mA cm^{-2})**	***FF*** **(%)**	**PCE (%)**
0 min	1.17	25.6	84.2	25.3
5 min	1.19	25.7	83.2	25.6
10 min	1.18	25.5	84.9	25.5
15 min	1.19	25.7	85.1	26.0
20 min	1.19	25.6	85.0	25.8
25 min	1.19	25.9	83.9	25.9
30 min	1.18	25.8	84.6	25.7
35 min	1.17	25.4	84.3	25.1
40 min	1.17	25.6	85.8	25.6
45 min	1.18	25.6	84.8	25.7
50 min	1.17	25.9	84.6	25.8
55 min	1.19	25.9	83.8	25.8
60 min	1.19	25.6	84.2	25.7
1,440 min	1.20	25.3	84.7	25.7
2,880 min	1.19	25.8	84.4	25.9
5,760 min	1.18	25.9	83.9	25.6
10,080 min	1.17	25.8	85.3	25.8
120 days	1.18	25.9	83.3	25.5

Table S5. Summary of perovskite ink shelf life reported recently.

**Ink composition**	**Storage condition**	**Shelf-life**	**Fabrication method**	**Device performance after storage**	**Reference**
γ-Valerolactone stabilized $FAPbI_3$-DMF/GVL ink	Sealed vials, T and RH are not specified	≥1 year (color & performance unchanged)	Spin coating	PSCs retained > 95% efficiency; 23% PCE obtained after 12-month storage	*Nat. Sustain.* 2023, 6, 1465
Dimethyl-ammonium formate stabilized $Cs_{0.05}(FA_{0.95}MA_{0.05})_{0.95}Pb(I_{0.95}Br_{0.05})_3$ DMF/DMSO ink	Ambient air 25–30 °C, 30–50% RH	≥ 14 days (no color/edge shift)	Spin coating	Ink with DMAFo unchanged; control ink red-shifted & discolored; DMAFo devices achieved 24.7% PCE	*Nat. Energy* 2024, 9, 536
Crown-ether stabilized $Cs_{0.04}(FA_{0.97}MA_{0.03})_{0.96}Pb(I_{0.97}Br_{0.03})_3$-DMF/DMSO ink	Glovebox, RT	Up to 120 days without detectable degradation	Spin coating	Crown-ether treated inks delivered 25.6% PCE and T95=1200 h	*Energy Environ. Sci.* 2024, 17, 7182
Piperazine-2-carboxylic acid dihydrochloride stabilized $Cs_{0.1}FA_{0.9}PbI_3$-DMF/DMSO ink	Ambient air, T and RH are not specified	20 days	Spin coating	Achieved 25.56% PCE after 20-day storage	*Adv. Funct. Mater.* 2025, 2505965
Sodium thiosulfate stabilized $CsMAFAPbI_3$ DMF/DMSO ink	Storage conditions are not clearly specified	> 60 days (no color change after 60-day light illumination)	Spin coating	Not studied	*J. Mater. Chem. A* 2025, d5ta01570g
Coordination-modulated $Cs_{0.1}FA_{0.9}PbI_3$-DMF/NMP ink	Ambient, 25°C and 50% RH	> 120 days	Blade coating	Cell PCE maintained at 25.5% after 120-day storage	This work

Table S6. Photovoltaic performance of 45 DMF/NMP perovskite mini-modules.

Module No.	Aperture area (cm^2)	V_{OC} (V)	I_{SC} (mA)	*FF* (%)	PCE (%)
1	12.6	7.22	49.6	81.3	23.3
2	12.6	7.23	50.0	81.8	23.5
3	12.6	7.22	50.0	81.3	23.4
4	12.6	7.20	49.8	78.3	22.4
5	12.6	7.09	50.5	77.3	22.1
6	12.6	7.12	50.6	78.9	22.7
7	12.6	7.11	50.5	78.4	22.4
8	12.6	7.10	51.8	77.8	22.9
9	12.6	6.93	50.7	77.3	21.5
10	12.6	6.99	50.5	78.5	21.9
11	12.6	7.02	50.6	78.9	22.2
12	12.6	7.03	50.7	78.8	22.5
13	12.6	7.23	48.2	81.6	22.6
14	12.6	7.10	48.4	76.6	20.9
15	12.6	7.26	48.2	76.4	21.2
16	12.6	7.23	48.1	81.5	22.5
17	12.6	7.17	48.7	77.8	21.5
18	12.6	7.25	47.7	82.5	22.6
19	12.6	7.25	47.9	81.7	22.5
20	12.6	7.23	48.2	81.5	22.5
21	12.6	7.24	48.2	82.1	22.8
22	12.6	7.23	48.3	80.9	22.4
23	12.6	7.25	47.6	80.2	21.9
24	12.6	7.24	48.1	77.2	21.3
25	12.6	7.22	47.4	78.6	21.3
26	12.6	7.15	48.6	80.3	22.1
27	12.6	7.17	48.8	81.4	22.5
28	12.6	7.15	48.6	80.6	22.2
29	12.6	7.19	48.4	80.6	22.2
30	12.6	7.09	50.5	77.4	22.1

31	12.6	7.13	50.6	78.9	22.7
32	12.6	7.12	50.5	78.4	22.5
33	12.6	7.20	49.9	78.3	22.4
34	12.6	7.23	49.6	81.3	23.2
35	12.6	7.23	49.7	81.5	23.3
36	12.6	6.97	50.6	78.8	22.1
37	12.6	6.92	51.0	76.9	21.5
38	12.6	7.20	50.8	75.5	21.8
39	12.6	7.18	50.3	76.2	21.8
40	12.6	6.95	50.7	77.3	21.6
41	63.2	14.1	123	73.6	20.3
42	63.2	14.2	125	78.3	21.9
43	63.2	14.1	122	74.7	20.1
44	63.2	14.4	123	78.5	21.8
45	63.2	14.5	123	77.3	21.8
